# Azaporphyrinoid‐Based Photo‐ and Electroactive Architectures for Advanced Functional Materials

**DOI:** 10.1002/adma.202514429

**Published:** 2025-10-21

**Authors:** Jorge Labella, Kobra Azizi, Dirk M. Guldi, Tomás Torres

**Affiliations:** ^1^ Department of Organic Chemistry Universidad Autónoma de Madrid Campus de Cantoblanco, C/ Francisco Tomás y Valiente 7 Madrid 28049 Spain; ^2^ Department of Chemistry and Pharmacy, FAU Profile Center Solar, Interdisciplinary Center for Molecular Materials (ICMM) Friedrich‐Alexander‐Universität Erlangen‐Nürnberg Egerlandstr. 3 91058 Erlangen Germany; ^3^ Institute for Advanced Research in Chemical Sciences (IAdChem) Universidad Autónoma de Madrid Madrid 28049 Spain; ^4^ IMDEA – Nanociencia C/ Faraday 9, Campus de Cantoblanco Madrid 28049 Spain

**Keywords:** azaporphyrinoids, photo‐ and electroactive architectures, phthalocyanines, solar cells, subphthalocyanines

## Abstract

Over the past two decades, a productive collaboration between the Torres and Guldi groups—at the Department of Organic Chemistry and the IAdChem Institute at Universidad Autónoma de Madrid, in collaboration with IMDEA Nanoscience, and the Interdisciplinary Center for Molecular Materials (ICMM) and FAU Profile Center Solar at Friedrich‐Alexander‐Universität Erlangen‐Nürnberg, respectively—has led to the development of a rich portfolio of azaporphyrinoid‐based photo‐ and electroactive architectures. These efforts have focused on the design and study of nanomaterials—including graphene and related 2D systems, smart stimuli‐responsive platforms, and nanostructured hybrids—with promising applications in energy, sustainability, electronics, and biomedicine. By combining expertise in synthetic chemistry and excited‐state dynamics, this partnership has enabled the construction of diverse donor–acceptor systems featuring phthalocyanines, subphthalocyanines, and related chromophores, covalently or supramolecularly integrated with fullerenes, carbon nanotubes, and graphene derivatives. In this conspectus, a focused overview of these contributions is presented, illustrating how such molecular ensembles have served as powerful platforms to unravel fundamental processes in light and charge management, including charge separation, energy funneling, transport, and recombination. Systematic structure–function studies have revealed key relationships that underpin photophysical behavior and support the rational design of high‐performance light‐harvesting systems. Beyond discrete molecules, significant advances have also been made in their integration into nanostructured devices and stimuli‐responsive materials for optoelectronic, photovoltaic, and biomedical applications.

## Graphical abstract

A long‐standing collaboration between the Torres and Guldi groups has yielded diverse azaporphyrinoid‐based donor‐acceptor nanohybrids with promising applications in solar energy conversion. This conspectus highlights key molecular platforms and structure‐function relationships that govern light and charge management, supporting the rational design of photo‐ and electroactive materials integrated into nanostructured devices.

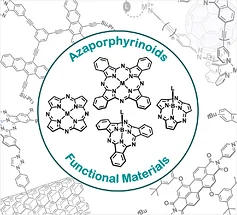

## 1 Introduction

Natural photosynthesis serves as a sophisticated blueprint for converting solar energy into chemical energy, relying on the coordinated interplay of photon absorption, energy transfer, and charge separation.^[^ ^1^ ^]^ Inspired by this biological paradigm, artificial photosynthesis and related energy conversion technologies aim to harness sunlight—a clean, abundant, and renewable energy source—to drive high‐energy chemical processes.^[^ ^2^ ^]^ Achieving such functionality in synthetic systems requires precise photon and charge management, through molecular and nanoscale components designed to mimic or outperform nature.

A key aspect of efficient photon management is the concept of *panchromaticity*—the ability to absorb light across a broad spectral range. In natural light‐harvesting complexes, spectral complementarity among chromophores ensures efficient energy funneling. In artificial systems, extending light absorption and enabling unidirectional energy transfer are key to maximizing photon utilization.^[^ ^3^ ^]^ Complementary strategies such as singlet fission (SF) and triplet–triplet annihilation (TTA) have emerged as powerful tools to manipulate photon energy, either by down‐converting a high‐energy photon into two excitons,^[^ ^4^ ^]^ or by combining two low‐energy excitons to emit one higher‐energy photon. These photophysical processes are crucial for enhancing the quantum efficiency of solar devices, with SF offering the potential to surpass the Shockley–Queisser limit of single‐junction solar cells, theoretically increasing it from ≈32% to ≈45.9%. Parallel to photon control, charge management—the efficient generation, separation, and transport of charge carriers—is equally critical. The Marcus theory provides a foundational framework to understand and predict charge‐transfer kinetics, highlighting the interplay between electronic coupling, reorganization energy, and thermodynamic driving forces.^[^ ^5^ ^]^

**Figure 1:**
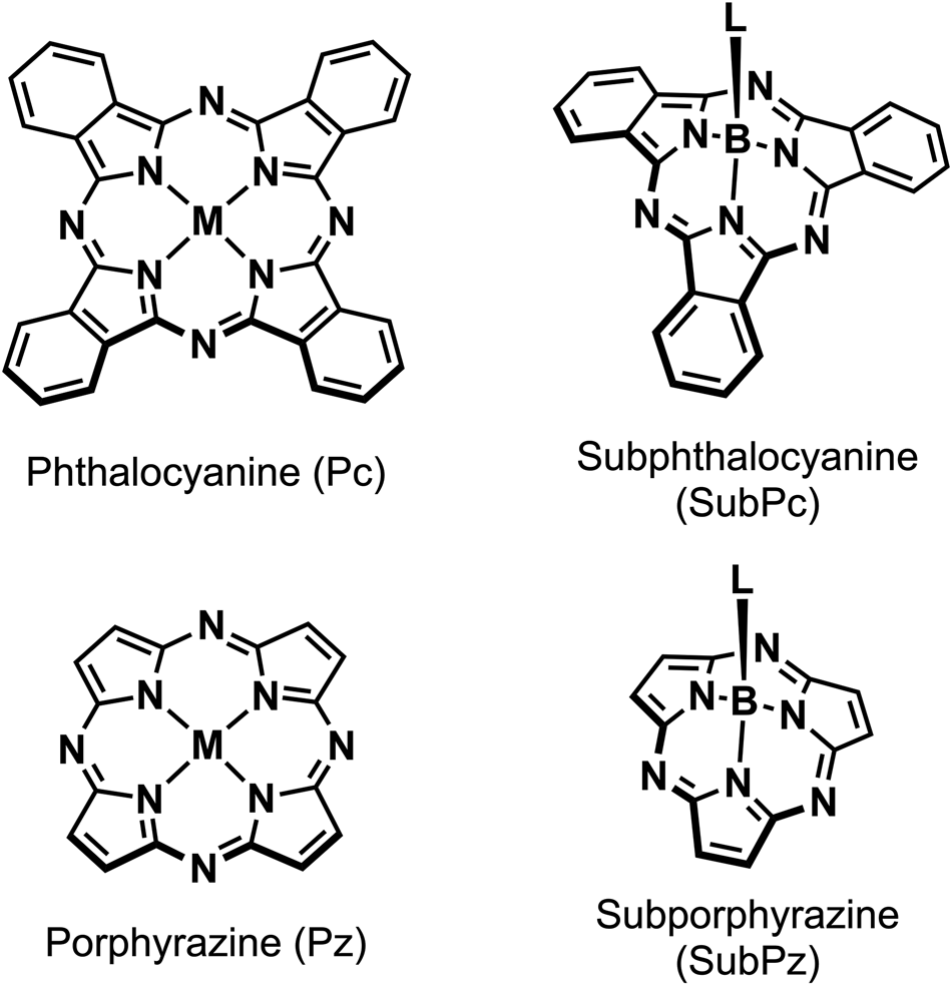
Molecular structure of the most representative azaporphyrinoids.

Azaporphyrinoids—a class of macrocyclic compounds primarily represented by phthalocyanines (Pcs) and subphthalocyanines (SubPcs), as well as their lower homologues, porphyrazines (Pzs) and subporphyrazines (SubPzs) (**Figure** **1**)— are particularly well‐suited for integrated photon and charge management. Pcs stand out for their remarkable chemical stability, synthetic tunability, and intense absorption in both the Soret (UV/blue) and Q‐band (red/NIR) regions. Their high molar extinction coefficients—typically above 200 000 m ^−1^ cm^−1^—enable efficient light harvesting over a broad spectral range, especially in the far‐red to near‐infrared region. Building upon this, our group has extended the library of Pcs to include heteroatom‐substituted analogues, such as antimony Pcs, with extinction coefficients exceeding 170 000 m ^−1^ cm^−1^ at ≈730–740 nm, and to Pzs, where the absorption profile can be substantially tuned via peripheral substitution, further demonstrating the potential of these systems for molecular photovoltaic and optoelectronic applications.^[^ ^6^ ^]^ These features make them ideal light harvesters and electron donors in molecular photovoltaics and related optoelectronic applications. On the other hand, SubPcs and SubPzs, nonplanar, 14 π‐electron aromatic analogues of Pcs, offer complementary advantages. Their bowl‐shaped geometry, strong fluorescence, high‐energy excited states (>2.0 eV), and low reorganization energies render them efficient photoactive scaffolds for energy conversion systems.^[^ ^7^, ^8^ ^]^ Furthermore, the SubPc core facilitates directional charge transfer and panchromatic absorption with minimal energy loss, making them promising components in organic solar cells, particularly as non‐fullerene acceptors.

Advanced carbon allotropes—fullerenes, carbon nanotubes (CNTs), and graphene—have revolutionized the field of molecular electronics and photovoltaics due to their exceptional optical, electronic, and mechanical properties.^[^ ^9^ ^]^ Fullerenes have long served as prototypical electron acceptors in donor–acceptor systems, enabling fundamental studies of charge separation and transport. CNTs and graphene, with their high surface area, tunable band structures, and amphoteric redox behavior, offer a versatile platform for both covalent and supramolecular functionalization, expanding the design space for hybrid photo‐ and electroactive materials.

Thus, over the past two decades, the collaborative efforts of the Guldi (Friedrich‐Alexander‐Universität Erlangen‐Nürnberg) and Torres (Universidad Autónoma de Madrid) groups have focused on integrating porphyrinoids with carbon nanostructures to develop next‐generation energy materials.^[^ ^10^, ^11^, ^12^, ^13^ ^]^

This interdisciplinary partnership combines synthetic molecular design with advanced photophysical and electrochemical characterization, spanning solution‐phase, thin‐film, and interfacial studies. The Torres group has pioneered the development of Pcs, Pzs, SubPcs, and SubPzs as versatile donor units, while the Guldi group has provided cutting‐edge insight into charge‐ and energy‐transfer dynamics through ultrafast spectroscopy and nanoscale assembly. Together, these efforts have resulted in a broad portfolio of donor–acceptor (D‐A) architectures based on azaporphyrinoids and carbon allotropes, optimized for panchromatic light harvesting and capable of undergoing charge separation and transport, energy transfer, or both. This review highlights the key achievements of this long‐standing collaboration, with a particular focus on the design principles, structure–property relationships, and potential application in molecular photovoltaics of azaporphyrinoid‐based photo‐ and electroactive materials.

It should be noted that this conspectus‐like review is not intended as a systematic review, but rather as a thematic overview focused on selected scientific contributions that illustrate key advances in the design and application of azaporphyrinoid‐based photo‐ and electroactive materials. Therefore, representative examples have been chosen based on their structural innovation, functional performance, and relevance to the collaborative work between the Torres and Guldi groups.

## 2 Covalent and Supramolecular Donor–Acceptor Architectures Based on Azaporphyrinoids

The rational design of D‐A systems based on porphyrinoids has enabled precise modulation of photoinduced charge separation and recombination processes. This section examines covalent and supramolecular strategies employing azaporphyrinoid scaffolds—Pcs, SubPcs, and their analogues—paired with electron‐accepting units. Key structural features such as linker geometry, supramolecular recognition motifs, and macrocycle curvature have been systematically exploited to enhance optical absorption, redox tunability, and excited‐state lifetimes. Emphasis is therefore placed on the interplay between molecular architecture and photophysical behavior in achieving high‐efficiency light‐harvesting assemblies for optoelectronic applications.

### 2.1 Phthalocyanine–Fullerene Donor–Acceptor Systems

The early development of Pc–C_60_ donor–acceptor conjugates laid the foundation for exploring the synergies between π‐extended chromophores and spherical electron acceptors. In this context, covalent and supramolecular strategies were employed to modulate donor–acceptor distance, orientation, and coupling strength. The use of functionalized fullerenes, tailored linkers, and π–π stacking or metal–ligand interactions enabled fine‐tuning of the photophysical response. Systematic studies revealed that electronic communication and radical ion pair lifetimes are highly dependent on the structural design, offering insights into the control of charge transfer processes.

**Figure 2:**
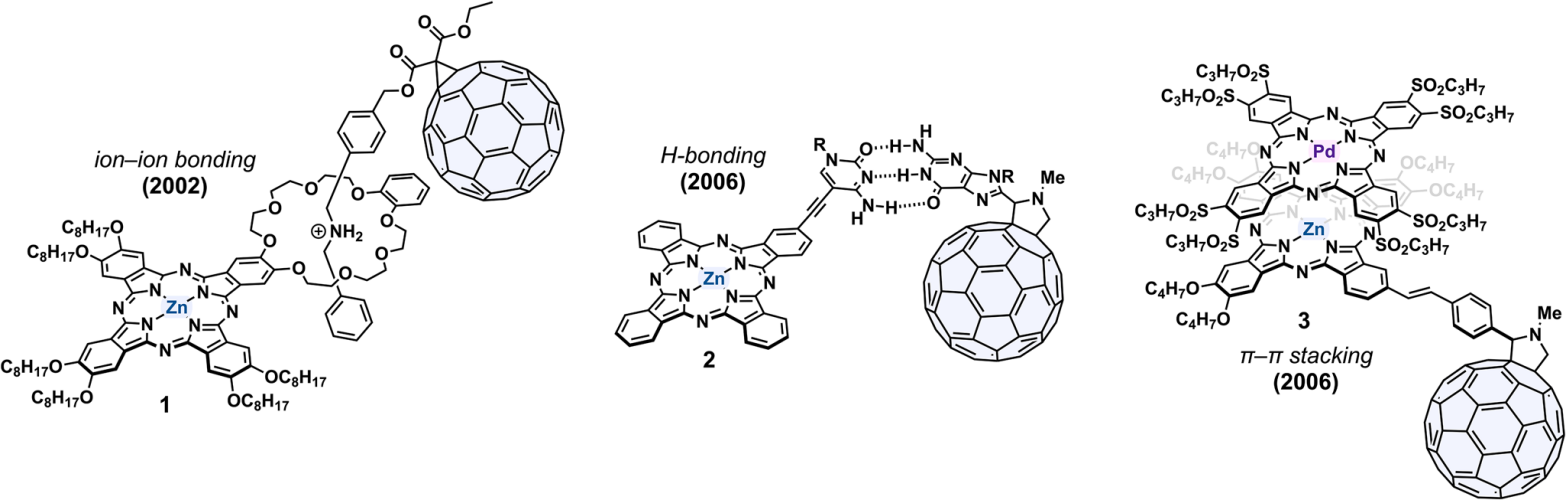
Core design principles for Pc–fullerene supramolecular donor–acceptor systems.

**Figure 3:**
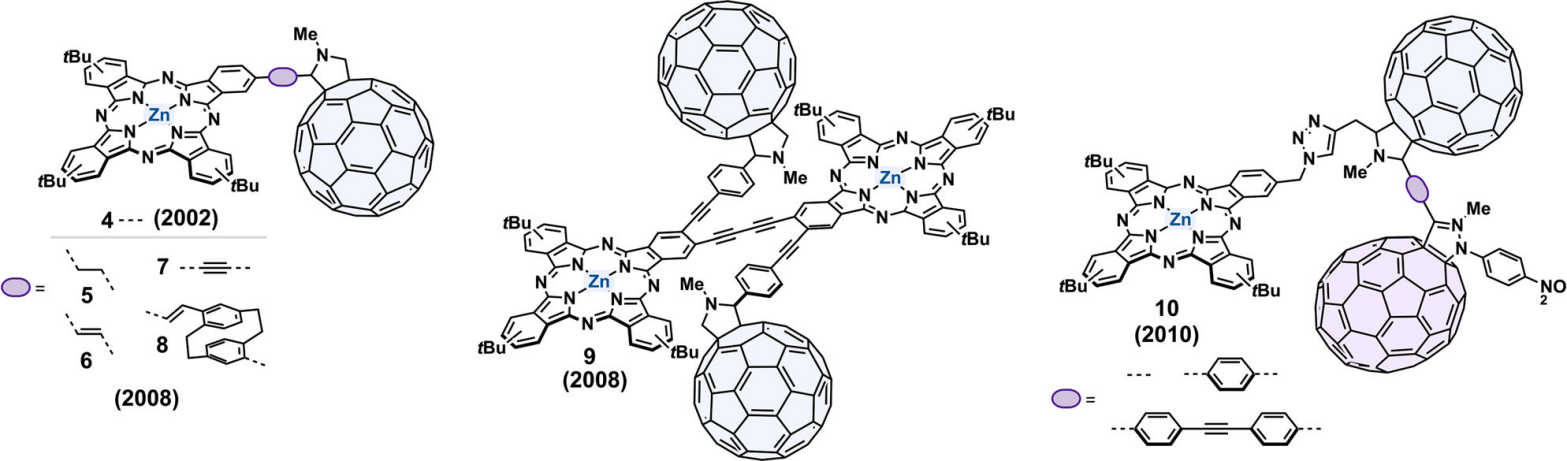
Representative covalent electron donor–acceptor systems based on Pc–fullerene conjugates.

**Figure 4:**
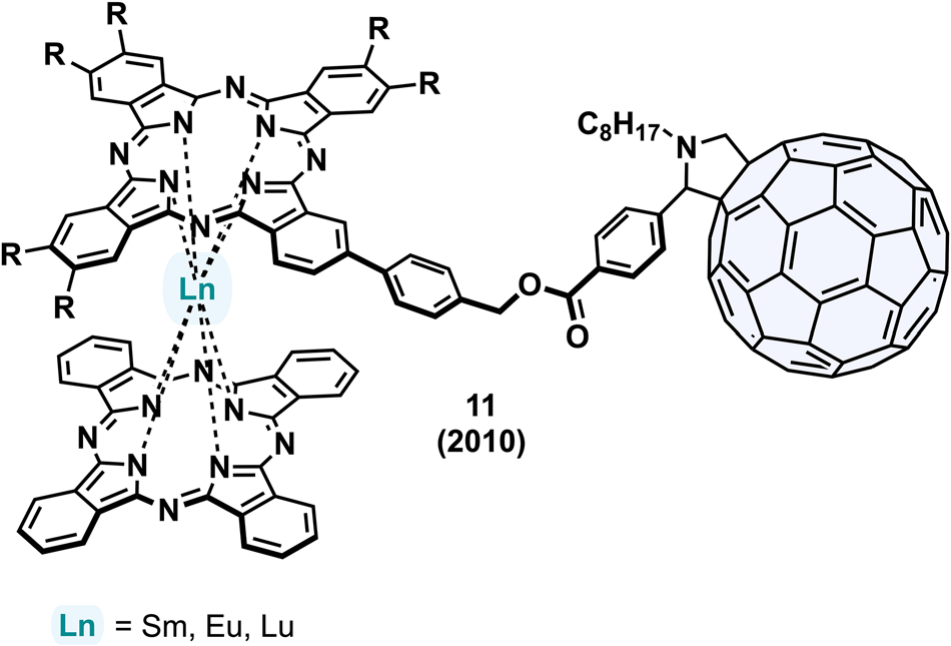
Molecular structures of double‐decker lanthanide(III) bis(phthalocyaninato)‐C_60_ dyads **11**.

**Figure 5:**
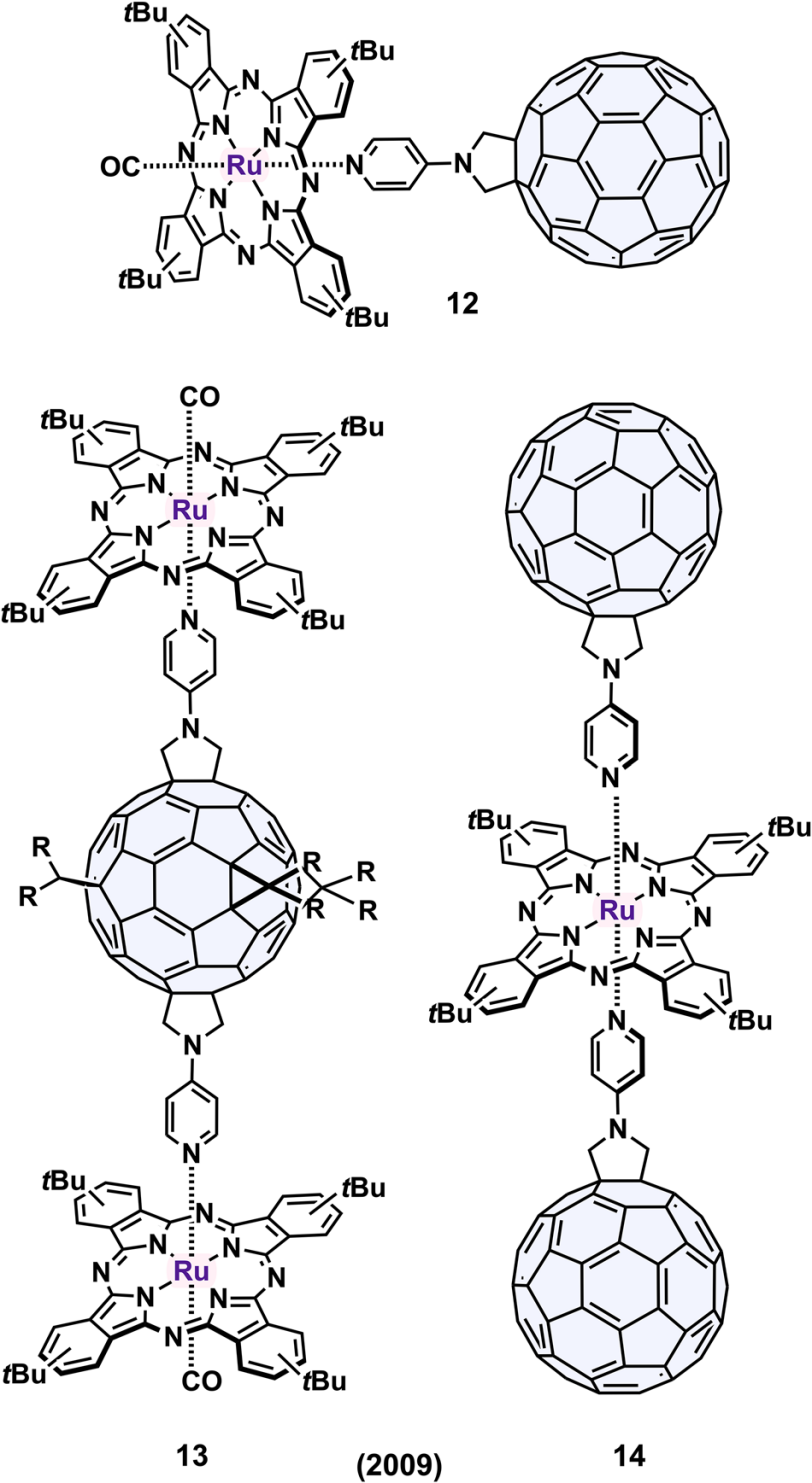
Molecular structures of supramolecular dyad Ru(II)Pc–C_60_ **12** and triads Ru(II)Pc_2_–C_60_ **13** and Ru(II)Pc–(C_60_)_2_ **14**.

**Figure 6:**
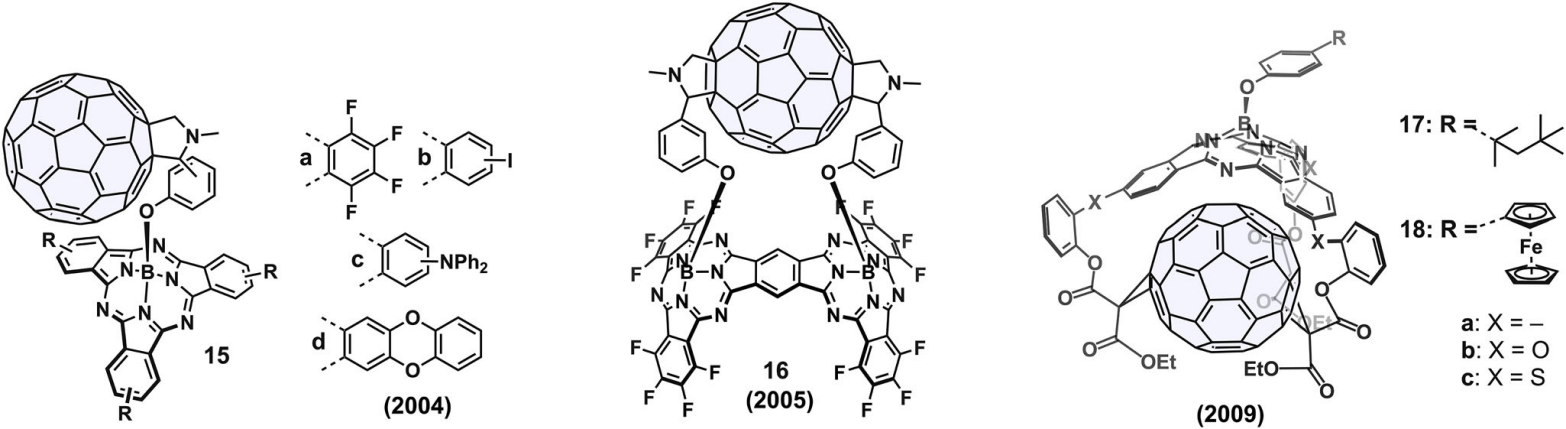
Representative covalent electron donor–acceptor systems based on SubPc–fullerene conjugates.

The collaboration between the Torres and Guldi groups, which began in 2002, was driven by a shared interest in exploring and combining the unique properties of Pcs and C_60_ fullerenes (**1–14**; **Figures** 2, 3, 4, 5, 6). This partnership firstly aimed to synthesize and study a simple Pc covalently linked to C_60_ (**4**; Figure 3), a project that would combine the strengths of both molecular structures.^[^ ^14^ ^]^ Pcs, known for their thermal and chemical stability, rich redox chemistry, and strong absorption in the red/near‐infrared (NIR) region of the solar spectrum, are ideal candidates for creating photoresponsive electron D‐A systems. In these systems, Pcs serve as efficient antennas, absorbing a significant portion of the solar spectrum, and as strong electron donors—or, to a lesser extent, as acceptors—upon photoexcitation. On the other hand, C_60_ fullerenes are celebrated for their excellent electron affinity, low reorganization energy in electron transfer processes, and superior charge transport capabilities. These properties make C_60_ an attractive electron acceptor for constructing photo‐ and electroactive systems.^[^ ^15^, ^16^ ^]^

At the time of their first joint publication, several Pc–C_60_ conjugates were already known.^[^ ^17^, ^18^ ^]^ However, it was in this seminal work that the photoinduced electron transfer between the photoexcited Pc and C_60_ was demonstrated for the first time. This breakthrough was achieved using transient absorption spectroscopy, which identified a transient feature in the NIR region around 1000 nm, confirming the formation of the one‐electron reduced form of C_60_ (i.e., C_60_^•‒^). This pioneering research paved the way for the development and study of numerous photoresponsive materials based on porphyrinoids, including Pcs, SubPcs, porphyrins (Pors), Pzs, and SubPzs. These materials were also combined with various electroactive units such as fullerenes, CNTs, graphene, perylene diimide (PDI), and 1,1,4,4‐tetracyanobuta‐1,3‐diene (TCBD).

Comparing covalent and noncovalent approaches, the latter offers several advantages. Noncovalent strategies allow for the creation of assemblies with tunable stability in both organic and aqueous solution, influenced by factors such as temperature, solvent polarity, and salt effects. This approach also enables the control of assembly and disassembly processes, thereby modulating the physicochemical properties of the systems. The Guldi and Torres groups successfully employed noncovalent interactions to construct D‐A ensembles using Pc and C_60_ units with complementary recognition motifs, as illustrated in Figure 2.

One strategy involved ammonium‐crown ether complexation to form Pc–C_60_ donor–acceptor ensembles. Zn(II)Pcs were modified with a single 24‐crown‐6 macrocycle at their peripheral positions, which were then threaded by alkylammonium fragments terminating in C_60_ **(1)**.^[^ ^19^ ^]^ Absorption and fluorescence titrations revealed interactions between the electron donor and acceptor, with *K*_ass_ of 10^4^ m ^‒1^ in dichloromethane. In the excited state, these D‐A ensembles led to the formation of radical ion pairs with lifetimes around 1 µs. Several years later, another approach involved modifying Zn(II)Pcs with one or four 18‐crown‐6 macrocycles, threaded by alkylammonium fragments ending in C_60_, showing an *K*_ass_ of 10^5^ m ^−1^ in methylcyclohexane, and radical ion pair lifetime of 104 µs in chlorobenzene^[^ ^20^ ^]^ While this strategy allows tuning the strength of ammonium‐crown ether complexation through temperature, ionic strength, and pH, it does not permit control over the relative distance and orientation between the electron donor and acceptor.

Hydrogen bonding interactions offer another method for creating biomimetic D‐A ensembles based on Pcs. By selecting appropriate hydrogen‐bonding motifs, the strength of the ensemble and the electronic coupling between the electron donor and acceptor can be finely tuned. On this basis, the Torres, Guldi, and Sessler groups utilized Watson–Crick hydrogen bonding interactions for the self‐assembly of a Pc–C_60_ D‐A ensemble (**2**).^[^ ^21^, ^22^ ^]^ This architecture was formed by complexing a fulleropyrrolidine bearing a guanosine with a cytidine‐substituted Pc. Absorption studies showed that the cytidine‐substituted Pc self‐assembled via cytidine/macrocycle interactions, forming aggregates that could be disrupted by adding the guanosine‐substituted C_60_. Fluorescence studies indicated a nonlinear decrease in the fluorescence of the cytidine‐functionalized Pc upon adding the guanosine‐substituted C_60_, suggesting charge separation upon D‐A ensemble formation. For this Pc–C_60_ D‐A complex, a *K*_ass_ of 2.6 × 10^6^ m ^−1^ and a radical ion pair state lifetime of 3 ns were determined in toluene. Using a similar strategy, Por/C_60_ D‐A ensembles were also prepared with a cytidine‐substituted Por.^[^ ^23^ ^]^ These ensembles exhibited radical ion pair lifetimes of up to 2 µs in dichloromethane, significantly longer than those of the Pc–C_60_ complex, likely due to the weaker *K*_ass_ of the Por/C_60_ complex (i.e., 5.1 × 10^4^ m ^−1^).

The Guldi and Torres groups have also explored the use of many other non‐covalent interactions to create Pc–C_60_ D‐A ensembles that can be assembled and disassembled using cooperative and/or orthogonal stimuli. For instance, the disassembly of a Zn(II)Pc dimer formed by head‐to‐tail metal‐ligand interactions of amidine‐functionalized Zn(II)Pc could be induced by the combined addition of a C_60_‐functionalized carboxylic acid and a phenothiazine (PTZ)‐substituted pyridine.^[^ ^24^ ^]^ The synergistic effects of Zn(II)Pc–pyridine metal–ligand interactions and amidine/carboxylic acid hydrogen bonding interactions led to the formation of the three‐component Zn(II)Pc dimer. In this complex, charge separation occurs from the photoexcited Zn(II)Pc to the electron‐accepting C_60_, followed by a charge‐shift from the electroactive PTZ to its coordinated Zn(II)Pc^•+^, ultimately generating a PTZ^•+^–Zn(II)Pc–C_60_^•‒^ radical ion pair state with an impressive lifetime of 7.5 µs in deoxygenated toluene, due to the spatial separation of the photogenerated electrons and holes.

In the realm of supramolecular chemistry, π‐stacking interactions have proven to be a powerful tool. A notable example involves the interaction between an electron‐deficient Pd(II) phthalocyanine (Pc) with eight alkylsulfonyl groups and a hexaalkoxy Zn(II)Pc linked to C_60_ (**3**). This interaction led to the formation of a 1:1 Pd(II)Pc–Zn(II)Pc–C_60_ complex in organic solution.^[^ ^25^ ^]^ The photogenerated radical ion pair state in these π‐stacked D‐A ensembles exhibited a lifetime of 475 ns, significantly longer than the 130 ns observed for the noncomplexed Zn(II)Pc‐C_60_ conjugate in THF. This demonstrates the stabilizing effect of supramolecular assembly on photogenerated charges. More recently, π‐stacking interactions have been harnessed to dramatically enhance the formation of Pc_2_‐C_60_ bisadduct enantiomers from a complex mixture of 130 bisadducts, increasing the yield by over 2800 times.^[^ ^26^ ^]^

Another supramolecular D‐A ensemble involved a sapphyrin, a pentapyrrolic expanded porphyrin, which recognized carboxylate anions on C_60_ derivatives functionalized with multiple carboxylate groups arranged dendritically.^[^ ^27^ ^]^ These noncovalent arrays exhibited sapphyrin‐to‐C_60_ electron transfer when irradiated with 387 nm light, resulting in lifetimes of ≈470 and 600 ps for the 1:1 and 1:2 sapphyrin‐C_60_ ensembles, respectively.

All in all, no general trend can be established between the type of noncovalent interaction and the radical pair lifetime, due to variations in solvent, molecular flexibility, and size. Nonetheless, the examples discussed highlight the rich supramolecular toolbox that azaporphyrinoids offer for engineering diverse and functionally tailored noncovalent D‐A architectures.

Turning to covalent Pc–C_60_ conjugates, early investigations primarily centered on the redox characteristics of Pcs^[^ ^28^ ^]^ and the proportion of Pc to fullerene components.^[^ ^29^ ^]^ For instance, our groups examined how varying the ratio of Pc to C_60_ affected the redox behavior and overall stability of the conjugates. Subsequent studies explored the nature of the linker connecting Pc and C_60_, including variations in length, rigidity, and conjugation.^[^ ^30^, ^31^ ^]^ The goal of these D‐A modifications was to understand how these changes would impact the dynamics of photoinduced energy and/or electron transfer processes, and in the case of electron transfer, the dynamics of photoinduced charge separation and recombination. For example, in a series of Pc–C_60_ conjugates (**5–7**; Figure 3), it was observed that variations in the conjugation and flexibility of the Pc–C_60_ linker significantly influenced the lifetime of the photogenerated radical ion pair states in anisole, with the longest lifetimes recorded for **7**, followed by **6** and **5** (i.e., 1200, 979, and 755 ps, respectively).^[^ ^32^ ^]^ In contrast, this trend was much less evident in toluene, THF, or benzonitrile. However, it is noteworthy that the alkyne‐based spacer consistently exhibits the longest lifetimes, suggesting it provides an optimal balance between rigidity and flexibility. Further studies refined the understanding of linker effects^[^ ^33^ ^]^ and revisited the Pc‐to‐fullerene ratio,^[^ ^34^ ^]^ examining how these factors influenced the photophysical properties and stability of the conjugates.

To investigate the impact of “pseudo conjugation” on the interaction between Pc and C_60_, a structurally rigid Pc‐C_60_ conjugate (**8**) was synthesized, where the two photo‐ and electroactive components were separated by a [2.2]para‐cyclophane spacer (Figure 3).^[^ ^35^ ^]^ This design resulted in a radical ion pair lifetime of 2600 ps in anisole, significantly longer than those observed for **5–7**. The para‐cyclophane spacer in **8** likely weakens the electronic coupling between Pc and C_60_ and increases the distance between the electron donor and acceptor compared to **5–7**. In addition, Pc–C_60_ conjugates with varying Pc‐to‐C_60_ ratios were prepared and analyzed. For instance, photophysical studies of compound **9**, which contains two Pcs and two C_60_ fullerenes, revealed relatively short radical ion pair lifetimes (Figure 3).^[^ ^36^ ^]^ The close proximity of Pc to C_60_ in this structure facilitates tight contact and through‐space deactivation dynamics, preventing the formation of long‐lived radical ion pair states. Comparative studies were also conducted on single‐decker versus double‐decker Pc structures.^[^ ^37^ ^]^ Single‐decker Pcs, with their simpler structure, were easier to synthesize and manipulate, whereas double‐decker Pcs offered enhanced electronic properties due to their stacked configuration.

Additional electroactive components such as Pors and oligo(*p*‐phenylenevinylene) (*o*‐PPV) were incorporated to enhance functionality.^[^ ^38^, ^39^, ^40^ ^]^ For example, incorporating Pors into the Pc–C_60_ conjugates improved light absorption and charge transfer capabilities, making them more suitable for photovoltaic applications.

A novel approach for unidirectional, long‐range charge transport within linear Pc–C_60_ conjugates was explored. This concept involved linking either a Zn(II)Pc or a Zn(II)Por to two fullerenes with different electron donor strengths, specifically C_60_ and C_70_, arranged in a precise sequence relative to Pc (**10**).^[^ ^41^ ^]^ The length and rigidity of the C_60_‐to‐C_70_ spacers were also varied. In these Pc–C_60_–C_70_ conjugates, a redox gradient was established from the excited‐state electron donor to C_60_ and then to C_70_, minimizing energy losses during the reductive charge shift between the two fullerenes. Excited‐state dynamics and computational deconvolution of transient absorption spectra provided evidence for cascades of short‐range charge‐transfer processes, including a reductive charge shift between the two electron‐accepting fullerenes. The kinetics of these processes depended on the nature and length of the respective spacer. Interestingly, a mediating state in the charge shift reaction at weak electronic couplings, involving a relationship between triplet‐triplet energy transfer and charge transfer, was also inferred. Notably, the longest ZnPc^•+^–C_60_/C_70_^−^ radical ion pair lifetime is observed for the system featuring the longest alkyne spacer (≈27 Å).

In addition to discrete covalent Pc–C_60_ conjugates, the Guldi and Torres teams, in collaboration with the Grubbs and Sariciftci groups, developed random polymeric materials containing photo‐ and electro‐active Pc and C_60_ pendant units. These polymers were synthesized via ring‐opening metathesis polymerization of Pc and C_60_ derivatives, both functionalized with a polymerizable norbornene moiety, using a Grubbs’ catalyst.^[^ ^42^ ^]^ Fluorescence studies on three different polymer batches with varying Pc‐to‐C_60_ ratios revealed quenching that strongly depended on the Pc‐to‐C_60_ content. Higher Pc‐to‐C_60_ ratios resulted in stronger quenching. For example, a polymer batch with a high Pc‐to‐C_60_ ratio exhibited significant fluorescence quenching, indicating efficient charge transfer between the donor and acceptor units. Notably, all polymers exhibited radical ion pair state lifetimes on the order of microseconds, suggesting a stabilizing effect of the photogenerated radical ion pair states when transitioning from discrete Pc–C_60_ molecular conjugates to polymeric Pc–C_60_ frameworks. Photovoltaic (PV) measurements indicated that the polymer backbone ensured close proximity of the Pc and C_60_ moieties, facilitating efficient charge separation but impeding charge transport to the electrodes, leading to moderate power conversion efficiency in the resulting devices. For instance, a polymeric Pc–C_60_ material demonstrated efficient initial charge separation but struggled with charge transport, resulting in lower overall device performance.

Another approach involved combining double‐decker lanthanide(III) bis(phthalocyaninato)s with C_60_ (**11**; Figure 4).^[^ ^43^ ^]^ The UV–vis absorption spectra of both components (double‐decker Pc and C_60_) overlapped, indicating the absence of ground‐state interactions. This lack of ground‐state electronic interactions was further supported by cyclic voltammetry studies, which showed superimposed features of double‐decker Pcs and C_60_. Electronic communication between the photo‐ and electroactive components in **11** was observed only upon photoexcitation at 387 nm, resulting in the formation of a long‐lived radical ion pair state that showed no notable decay within the 3 ns time window of the femtosecond experiments, suggesting a lower limit for the lifetime of >3 ns in toluene.

Photoinduced charge separation was achieved in Sc_3_N@*I_h_*‐C_80_‐based D‐A conjugates, where the endohedral metallofullerene functioned as the electron acceptor.^[^ ^44^ ^]^ Two isomeric conjugates, each bearing triphenylamine (TPA) as the electron donor, were compared with their C_60_ analogues to assess the impact of molecular design.^[^ ^45^ ^]^ Linkage via the pyrrolidine nitrogen resulted in radical ion pair states with longer lifetimes and greater thermal stability than the 2‐substituted isomer, with these effects amplified in Sc_3_N@*I_h_*‐C_80_ derivatives. Substitution of C_60_ with Sc_3_N@*I_h_*‐C_80_ significantly prolonged the radical pair lifetimes, establishing the metallofullerene as a superior acceptor. These structure–function relationships then guided the development of porphyrinoid–fullerene architectures incorporating Y_3_N@C_80_ and Sc_3_N@C_80_,^[^ ^46^ ^]^ despite persistent limitations such as low functionalization yields and reduced product stability, which restricted comprehensive photophysical and electrochemical analysis.

Utilizing metal–ligand interactions has proven to be an effective method for creating Pc–C_60_ D‐A ensembles. This is achieved by coordinating a pyridyl‐substituted C_60_ fulleropyrrolidine with various Zn(II)Pcs, such as BODIPY‐substituted Zn(II)Pcs,^[^ ^47^ ^]^ NIR‐absorbing Zn(II)azulenocyanines,^[^ ^48^ ^]^ or Zn(II)Pcs fused^[^ ^49^ ^]^ or covalently linked to Pors.^[^ ^50^, ^51^ ^]^

This strategy is particularly useful to address the chemical stability issues encountered in the synthesis of covalent Pc‐EMFs, and therefore D‐A supramolecular ensembles based on Pcs and endohedral metallofullerenes (EMFs) were prepared and studied by the two research groups. To this end, a N‐pyridyl‐substituted Sc_3_N@*I_h_*‐C_80_ was axially coordinated to either electron‐rich or electron‐deficient Zn(II)Pcs through zinc‐pyridyl, metal‐ligand coordination.^[^ ^52^ ^]^ Photophysical studies conducted on these D‐A ensembles revealed clear evidence of charge separation occurring in the photoexcited Zn(II)Pc‐to‐Sc_3_N@*I_h_*‐C_80_ system. Remarkably, when paired with an electron‐rich Zn(II)Pc, Sc_3_N@*I_h_*‐C_80_ acts as an electron acceptor, facilitating charge separation. On the other hand, when paired with an electron‐deficient Zn(II)Pc, Sc_3_N@*I_h_*‐C_80_ acts as an electron donor, transferring electrons to the Pc. This versatility in electron transfer behavior makes Sc_3_N@*I_h_*‐C_80_ a fascinating subject for designing efficient D‐A systems with tailored functionalities.

To enhance the kinetic stability of Pc‐C_60_ D‐A ensembles, the central zinc atom in the Pc was replaced with ruthenium. This led to the creation of Ru(II)Pc–C_60_ (**12**) and Ru(II)Pc_2_–C_60_ (**13**) (Figure 5).^[^ ^53^ ^]^ The synthesis involved treating a (*t*Bu)_4_‐Ru(II)Pc, which has an axial carbonyl group at one of its two axial Ru(II) coordination sites, with either a monopyridyl‐functionalized C_60_ (resulting in **12**) or a bispyridyl‐functionalized C_60_ with two pyridyl units in a trans‐1 arrangement (resulting in **13**). In addition, the Ru(II)Pc–(C_60_)_2_ triad **14** was synthesized by reacting a monopyridyl‐functionalized C_60_ with a (*t*Bu)_4_‐Ru(II)Pc that has two labile benzonitrile molecules at its axial positions. Electrochemical studies on compounds **12–14** indicated electronic coupling between Pc and C_60_ in the ground state. From a photophysical perspective, using Ru(II)Pcs instead of Zn(II)Pcs, which have a high‐lying triplet excited state, offers significant advantages. Specifically, the unwanted and energy‐wasting charge recombination was effectively slowed down by pushing it into the Marcus inverted region. As a result, the lifetimes of the radical ion pair states were extended to hundreds of nanoseconds when using monopyridyl‐functionalized C_60_ in compounds **12** and **14**. In **13**, which features a unique hexakis‐C_60_ functionalization, a cathodic shift in the reduction potential was observed, increasing the energy of the radical ion pair state. However, the triplet excited‐state energy localized on the Ru(II)Pc was insufficiently high, leading to rapid deactivation of the radical ion pair state.

In collaborative research, Pc–C_60_ ensembles were synthesized using metal–ligand coordination to investigate the impact of distance between the electron‐donating Pc and electron‐accepting C_60_ on long‐range electron transfer. The focus was on reorganization energy and attenuation factor.^[^ ^54^ ^]^ Three pyridyl‐substituted C_60_ fulleropyrrolidines were synthesized, varying the distance between C_60_ and the pyridine ring by modifying the length of the *o*‐PPV spacers. In these D‐A ensembles, radical ion pair state lifetimes increased from 0.4 to 86.6 ns as the separation increased from 8.8 to 29.1 Å. Kinetic analysis revealed a relationship between radical ion pair state lifetimes and i) electronic coupling elements, ranging from 132 to 1.2 cm^‒1^, and ii) reorganization energies of electron transfer, varying from 0.43 to 0.63 eV. In addition, an attenuation factor of 0.27 Å^‒1^ was determined for the *o*‐PPV spacers. Similar results were obtained when Zn(II)Pc was replaced with Zn(II)Por.^[^ ^55^ ^]^ A bisfullerene structure, where two C_60_ fullerenes are connected by a bis(dipyrrinato)zinc complex bridge, was also developed in collaboration with another research group.^[^ ^56^ ^]^ This strongly coupled D‐A ensemble demonstrated efficient charge separation upon laser photoexcitation at 480 nm.

Going one step further, an innovative binding strategy was employed by combining multipoint hydrogen bonding and metal–ligand complexation to create a D‐A supramolecular ensemble. The resulting ensemble featured a cyanuric acid‐substituted Zn(II)Por, a PDI unit with a Hamilton receptor, and a pyridine‐substituted C_60_.^[^ ^57^ ^]^ This tricomponent architecture exhibited panchromatic properties due to the complementary optical absorption of Por and PDI. Photophysical studies revealed that selective photoexcitation of the PDI in the hydrogen‐bonded Por/PDI system triggers energy transfer from the PDI singlet excited state to the Zn(II)Por. In contrast, the pyridine‐substituted C_60_ and Zn(II)Por self‐assemble through axial complexation, leading to a radical ion pair state with a lifetime of 1.0 ns. Time‐resolved measurements on the PDI/Zn(II)Por/C_60_ system showed that the initial energy transfer from PDI to Zn(II)Por is followed by electron transfer from Zn(II)Por to C_60_, resulting in a radical ion pair state with a lifetime of 3.8 ns.

Cation‐crown ether and metal‐ligand interactions were also utilized to prepare multicomponent D‐A ensembles based on Pc and C_60_. Zn(II)Pcs decorated with four crown ethers at their peripheral positions were co‐facially dimerized by adding rubidium cations through cation‐crown ether interactions. These Pc dimers were further complexed at their outer axial positions with pyridyl‐functionalized C_60_ or PDI through metal–ligand interactions.^[^ ^58^ ^]^ Transient absorption experiments on the rubidium‐complexed Pc dimers, in the absence of C_60_, revealed intramolecular electron transfer, forming a Zn(II)Pc^•+^/Zn(II)Pc^•‒^ radical ion pair state. Upon metal–ligand complexation with pyridyl‐functionalized C_60_ or PDI, the formation of Zn(II)Pc_2_^•+^/PDI^•‒^ or Zn(II)Pc_2_^•+^/C_60_^•‒^ radical ion pair states were observed, with the latter showing the longest lifetime (5.1 ns vs 3.4 ns in *o*‐dichlorobenzene).

Synthesizing and characterizing D‐A conjugates in aqueous media presents a significant challenge. To address this, a photosynthetic model system based on Pc–C_60_ was developed using electrostatic interactions.^[^ ^59^ ^]^ A tetracationic Zn(II)Pc bearing pyrenes was reported to organize into linear supramolecular polymers that bundle into crystalline rod‐like structures in a 95:5 water/dimethylsulfoxide mixture. When a negatively charged C_60_ derivative was immobilized onto these Zn(II)Pc assemblies, photogenerated charges were funneled from the aggregates to the electron‐accepting C_60_ units, resulting in long‐lived charge‐separated (CS) states. In another collaborative effort, π‐stacking and hydrophilic‐hydrophobic interactions were utilized to promote the self‐assembly of covalently linked and amphiphilic Zn(II)Pc‐C_60_ in water, forming uniform, micrometer‐long nanorods.^[^ ^60^ ^]^ Transient absorption studies on this conjugate showed remarkable stabilization of the photogenerated radical ion pair state lifetime, increasing from ≈3 ns to 1.4 ms for the self‐assembled nanotubules compared to a Zn(II)Pc‐C_60_ lacking the hydrophilic ammonium unit and unable to self‐assemble.

In summary, Pc–C_60_ D‐A systems demonstrated how structural modifications dictate charge separation dynamics, providing a versatile platform for tuning optoelectronic behavior.

### 2.2 Subphthalocyanine–Fullerene Donor–Acceptor Systems

SubPcs are analogues of Pcs that consist of three diiminoisoindoline units N‐fused around a central boron atom.^[^ ^7^, ^8^ ^]^ These macrocycles possess an extended electronic structure with a 14‐π‐electron aromatic core and a curved geometry, making them attractive for constructing both covalent and noncovalent D‐A systems. SubPcs have demonstrated their potential in molecular PVs, being utilized in both bulk and planar heterojunction configurations showcasing their efficiency as light‐harvesting and electron‐accepting materials in non‐fullerene organic solar cells.^[^ ^61^, ^62^, ^63^, ^64^ ^]^

**Figure 7:**
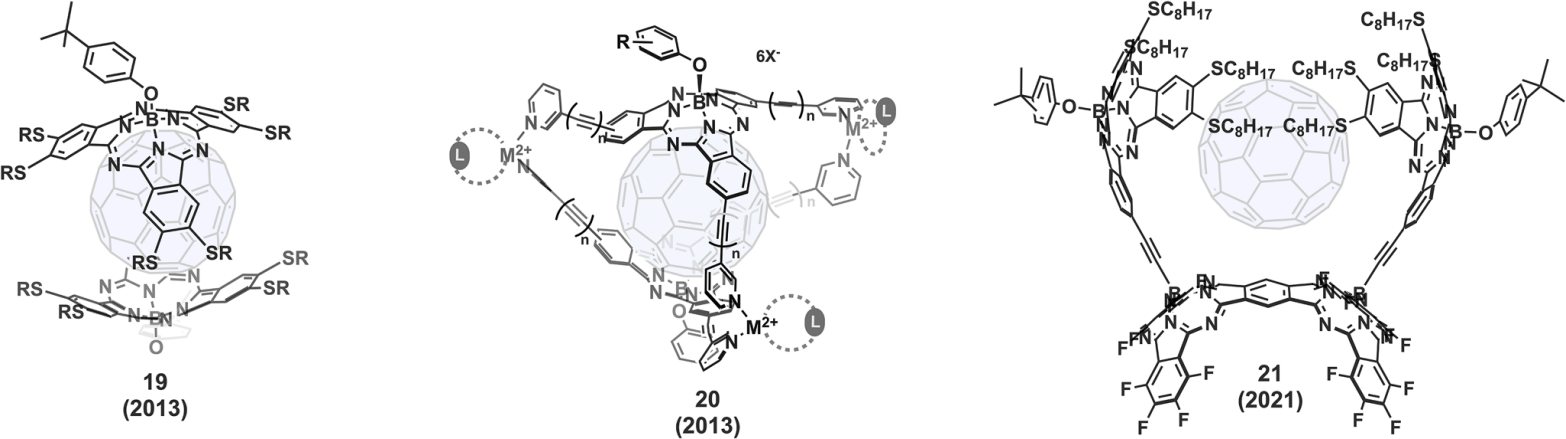
Representative supramolecular electron donor–acceptor systems based on the concave–convex interaction between SubPcs and fullerenes.

SubPcs introduce unique opportunities for D‐A system design due to their bowl‐shaped curvature and axial functionalization capability. Their electronic and steric properties enable precise control over fullerene interactions, whether through covalent conjugation or supramolecular complexation. Strategies involving axial and peripheral substitution, π‐surface complementarity, and topological isomerism have been implemented to modulate energy and electron transfer pathways. These design principles support the construction of photoactive materials with directional charge flow and tunable excited‐state behavior (**15–21**; Figures 6 and **7**).

Functionalization of SubPcs can occur at either the peripheral positions or the axial position (i.e., at the boron atom). Peripheral functionalization involves substituting the isoindoles, similar to most Pc‐based D‐A systems. However, SubPcs can also be easily functionalized covalently at the axial position.^[^ ^65^ ^]^ This axial functionalization offers two main advantages: it simplifies the preparation process by avoiding the need for peripherally substituted SubPcs, including unsymmetrical ones, and it preserves the electronic properties of the central core from the influence of axial substituents, as the boron atom acts as an electronic barrier.

Similar to Pcs, C_60_ fullerenes have been incorporated into SubPc‐based D‐A systems. The first SubPc‐C_60_ conjugate was reported in 2002.^[^ ^66^ ^]^ Subsequently, a series of four different SubPc‐C_60_ dyads (**15a–d**) were synthesized and studied (Figure 6).^[^ ^67^, ^68^ ^]^ While the linker between SubPc and C_60_ remained constant, the peripheral substituents on the SubPcs were varied, ranging from electron‐withdrawing fluorine or iodine to electron‐donating alkoxy or amine groups. Systematic variation of the peripheral substituents in dyads **15a–d** enabled fine‐tuning of the SubPc oxidation potential (shift of ≈200 mV from **15a** to **15d**), while the fullerene reduction remained unchanged. This modulated the energy of the SubPc^•+^–C_60_^•^^−^ CS state, influencing its competition with energy transfer deactivation. In nonpolar solvents, dyads **15a–15c** underwent efficient singlet–singlet energy transfer to C_60_, followed by intersystem crossing and triplet–triplet energy transfer back to SubPc, whose triplet‐state energy (≈1.45 eV) was determined by phosphorescence. In contrast, reduced redox gaps and increased solvent polarity favored electron transfer. For example, **15c** in benzonitrile yielded a CS state with a 0.65 ns lifetime, while **15d**, with a more stabilized CS state, exhibited lifetimes two orders of magnitude longer, consistent with the Marcus inverted region. These results highlight how peripheral substitution governs the balance between EnT and PET in SubPc–C_60_ dyads.

A similar strategy was followed to use fused SubPc‐dimers (i.e., two SubPcs sharing a benzene ring)^[^ ^69^, ^70^ ^]^ as donor systems, revealing different electronic properties compared to monomeric SubPcs due to their topological isomeric forms (*syn* and *anti*). A notable example is the D‐A conjugate **16** where a perfluorinated *syn*‐SubPc dimer was doubly and covalently linked to C_60_ (Figure 6).^[^ ^71^ ^]^ This synthesis involved a double 1,3‐cycloaddition of two azomethine ylide moieties, formed in situ at the axial positions of the *syn*‐SubPc dimer, to C_60_, known as a double Prato reaction. Subsequent theoretical studies at the semiempirical level provided insights into the possible constitutional isomers of this D‐A conjugate and their likely distributions.^[^ ^72^ ^]^ Photophysical investigations, using nanosecond and femtosecond experiments, revealed a cascade of energy transfer events, with excitation energy oscillating between the SubPc dimer and C_60_ following the initial photoexcitation of the SubPc dimer.

As previously mentioned, fullerenes are typically excellent electron acceptors, but their role as electron donors is less common. In 2015, SubPc‐C_60_ D‐A conjugates were created using a 1,3‐dipolar cycloaddition reaction with electron‐deficient SubPcs that had electron‐withdrawing fluorine or pentylsulfonyl substituents.^[^ ^73^ ^]^ This approach aimed to facilitate photoinduced oxidation of C_60_ with low reorganization energies. Time‐resolved spectroscopy revealed unambiguous evidence of an ultrafast oxidative electron transfer from C_60_ to the photoexcited SubPc. This represented the first documented case of intramolecular oxidation of C_60_ within a D‐A conjugate achieved exclusively through photoexcitation.

The development of more intricate, covalently linked SubPc‐C_60_ D‐A conjugates advanced with the creation of **17**, which features two complementary π‐surfaces: a concave SubPc and a convex C_60_ (Figure 6).^[^ ^74^ ^]^ This strategy utilized SubPcs with three pendant malonate groups, which reacted with C_60_ through a triple Bingel–Hirsch reaction. This reaction exhibited high regioselectivity and full diastereoselectivity due to the semi‐rigid nature of the tethers. Photophysical and electrochemical studies on dyads **17a–c** revealed how the nature of the spacer modulates the balance between energy and electron transfer. In **17a**, which features the shortest D‐A distance due to a rigid phenyl bridge, strong ground‐state electronic coupling between SubPc and C_60_ was observed, evidenced by redox potential shifts and perturbed absorption profiles. Upon photoexcitation, this tight interaction favors rapid charge separation (*k*_CS_ ≈ 1.4 × 10^11^ s^−1^) leading to a long‐lived radical ion pair (97 ns in THF), stabilized by partial delocalization of the hole toward the axial phenoxy group. In contrast, the increased flexibility and donor–acceptor distance in **17b** and **17c** (phenoxy and thiophenoxy spacers, respectively) weaken ground‐state interactions, and photodeactivation proceeds primarily via singlet–singlet energy transfer (*k*_ET_ ≈ 1.5 × 10^11^ s^−1^), intersystem crossing on the fullerene, and triplet–triplet back‐transfer to SubPc. This results in SubPc triplet formation (1.45 eV), rather than radical pair generation. These results underscore the critical role of orbital overlap and rigidity in tuning the mechanistic outcome of photoexcited SubPc–fullerene conjugates.

To further illustrate the influence of π–π distance and axial ligand on photophysical behavior, C_60_‐SubPc‐Fc conjugates **18a** and **18b** were synthesized (Figure 6).^[^ ^75^ ^]^ These were designed with ferrocene (Fc) moieties attached at the SubPc axial position via a phenoxy spacer, while C_60_ was rigidly positioned close to the concave face of the macrocycle through a threefold C_3_‐symmetrical anchoring. The primary difference between **18a** and **18b** lies in the length of the triple tether, which affects the regioselectivity of the trisaddition Bingel–Hirsch reaction and the distance between the concave–convex π–π surfaces of SubPc and C_60_. A comparative photophysical study between **18a** and **18b** was conducted. In **18a**, a direct C─C bond affords a shorter π–π distance (3.25–3.30 Å), whereas in **18b**, an ether linkage (C─O─C) increases the separation to 3.5–3.6 Å. Despite comparable HOMO–LUMO gaps, only **18a**—with closer proximity and stronger orbital overlap between the concave SubPc and the fullerene—undergoes efficient, photoinduced multistep electron transfer, ultimately yielding a long‐lived, spatially separated C_60_˙^−^/Fc˙⁺ radical ion pair with a lifetime of ≈0.1 ms. In contrast, the weaker ground‐state interaction in **18b** suppresses this charge separation pathway, underscoring how subtle structural variations in π–π stacking distances can dictate excited‐state reactivity in SubPc‐based D‐A systems.

The same research groups reported the synthesis of a series of SubPc‐La_2_@C_80_ donor–acceptor conjugates, which were characterized using cyclic voltammetry, absorption, fluorescence, and femtosecond‐resolved transient absorption spectroscopy. Interestingly, the strong electron‐donating nature of La_2_@C_80_ was crucial for driving intramolecular electron transfer in SubPc‐La_2_@C_80_ upon photoexcitation.^[^ ^76^ ^]^

In most cases where C_60_ is covalently linked to the axial position of a SubPc, energy transfer typically occurs after photoexcitation. However, when C_60_ is attached to face the concave part of SubPc, the distance between the π–π surfaces determines whether electron or energy transfer occurs.^[^ ^69^ ^]^ Interactions between C_60_ and the concave face of SubPcs can occur even if the carbon nanostructure and the subporphyrinoid are noncovalently linked through shape complementarity of their π–π surfaces, provided the host/guest binding constants are sufficiently high.

Keeping this idea in mind, a series of SubPcs with systematically varied lengths of six peripheral alkylthio units (**19**; Figure 7) were synthesized to study their interactions with C_60_ or C_70_ in organic solution and to determine the structural factors influencing fullerene encapsulation.^[^ ^77^ ^]^ Titration and Job's plot experiments provided quantitative data on the stoichiometry and strength of the complexation. Hydrophobic interactions, complemented by π–π interactions, were identified as the primary driving forces behind the 2:1 complexation between two SubPcs and one C_60_ or C_70_, with binding constants as high as 10^5^ m ^‒1^. The substantial absorption cross‐sections of SubPcs across the visible spectrum facilitate unidirectional energy transfer, funneling the excited‐state energy from the SubPcs to C_60_ or C_70_.

Building upon these results, a systematic and quantitative study was conducted to investigate the interactions between SubPc‐based capsules and C_60_ in organic solution. The goal was to understand the structural factors necessary for controlled C_60_ encapsulation and the resulting host/guest characteristics. Four different M_3_L_2_ capsules were synthesized through the metal‐induced dimerization of two C_3_‐symmetry SubPcs bearing three peripheral 3‐pyridyl units (**20**), and their ability to complex with C_60_ was examined.^[^ ^78^ ^]^ SubPcs, with their curved structure and synthetic versatility, are ideal for constructing functional homodimeric capsules. The high degree of symmetry in these cages resulted from chiral self‐discrimination between the enantiomers of the starting SubPc during the self‐assembly process, as C_3_‐symmetry SubPcs consist of a racemic mixture of two enantiomers.^[^ ^79^ ^]^

Research has demonstrated that metallo‐cages **20** can form 1:1 complexes with C_60_ and C_70_, along with their soluble PCBM‐type derivatives in dichloromethane. The π–π interactions between C_60_/C_70_ and SubPcs were analyzed by measuring the binding constants and the stoichiometry of the resulting complexes. Furthermore, time‐resolved photophysical studies were conducted to explore the nature of the host/guest interactions. Pump‐probe experiments revealed that photoexcitation of the SubPc‐based capsules triggers a swift transfer of singlet excited‐state energy to C_60_. Following this, C_60_ undergoes intersystem crossing before the energy is transferred back to the SubPc‐based capsules. Overall, the transfer of triplet excited‐state energy is relatively slow before it deactivates to the ground state.

In a subsequent work, SubPcs decorated peripherally with either six or three Fc units were also synthesized.^[^ ^80^ ^]^ As anticipated, the peripherally‐linked Fc units significantly impacted the electronic properties of SubPcs, causing substantial red‐shifts of the macrocycle Q‐bands in the range of 44–70 nm. The SubPc–Fc conjugates host C_60_ and form cocrystallates in the solid state. Specifically, both convex–convex and concave–convex interactions enable a unique linear arrangement in the crystalline state. However, the strong perturbation of the electronic structure caused by the Fc moieties leads to rapid deactivation of the excited states, preventing either energy or electron transfer. Developing new chromophoric receptors for binding C_60_ or C_70_ is fundamental to the research for improved fullerene‐based organic photovoltaics.

The quest for advanced chromophoric receptors capable of binding C_60_ or C_70_ is fundamental to enhancing fullerene‐based organic photovoltaics. Recently, a novel SubPc‐based conjugate, referred to as **21**, was synthesized. This conjugate features an electron‐deficient perfluorinated SubPc fused dimer, which is axially connected to two electron‐rich thioether‐substituted SubPcs through a double Sonogashira reaction (Figure 7).^[^ ^81^ ^]^ This structure is meticulously designed to efficiently complex with C_60_ or C_70_. The two electron‐rich SubPcs are firmly attached to the π‐extended convex surfaces of the SubPc dimer, creating a multicomponent system with a tweezer‐like topology that encapsulates C_60_ or C_70_ within its inner pseudo‐cavity, exhibiting a binding constant of ≈10^5^ m ^‒1^. Moreover, the photoactive properties of both the monomeric and dimeric SubPc units enabled an in‐depth physicochemical analysis. Upon photoexcitation, a series of energy and electron transfer processes are initiated, whether C_60_ or C_70_ is present or not. In polar solvents, the excited state leads to the formation of an intramolecular SubPc^•+^–SubPc dimer^•‒^ radical ion pair. When C_60_ or C_70_ is present, this state undergoes an unusual charge shift, resulting in the formation of an intermolecular SubPc^•+^–C_60_^•‒^ or SubPc^•+^–C_70_^•‒^ radical ion pair due to the close proximity between the fullerene and the electron‐rich SubPc “bucky‐catcher.”

In conclusion, the axial and concave nature of SubPcs has been exploited to develop structurally diverse SubPc–C_60_ conjugates with finely controlled charge transfer properties.

### 2.3 Azaporphyrinoid‐Based Donor–Acceptor Systems with Non‐Fullerene Acceptors

Efforts to expand the functionality and versatility of porphyrinoid‐based D‐A systems have led to the exploration of non‐fullerene electron acceptors, aiming to overcome the intrinsic limitations of traditional fullerene derivatives.^[^ ^82^, ^83^, ^84^, ^85^ ^]^ Historically, a substantial portion of the collaborative work by Guldi and Torres centered on Pc‐ and SubPc‐based D‐A systems where fullerenes such as C_60_ or C_70_ acted as electron acceptors. These systems benefited from the unique electron‐accepting capabilities of fullerenes but suffered from drawbacks such as limited absorption in the visible spectrum and restricted tunability of frontier orbital energies due to the inert nature of the fullerene cage. The new non‐fullerene acceptors thus employed covalent and supramolecular designs to enhance spectral coverage, redox tunability, and long‐lived CS states. On this basis, SubPcs, SubPzs, and hemiporphyrazines were coupled to NFAs such as PDIs, TCBDs, and cyclopenta‐fused aromatics. Emphasis was placed on modulating electronic coupling, excited‐state dynamics, and spatial organization, expanding the functional landscape of porphyrinoid materials in optoelectronics.

In response to these challenges, the two groups systematically explored multicomponent assemblies in which porphyrinoid and azaporphyrinoid units—ranging from Pcs and SubPcs to Pors, Pzs, and SubPzs—were combined with non‐fullerene acceptors. Structurally tunable porphyrinoids offer broad and intense absorption across the UV–vis–NIR range, along with high thermal and photochemical stability. Their synthetic accessibility enables fine control over energy levels, thin‐film morphology, and supramolecular organization. These features support their integration into both organic and perovskite solar cells. For comprehensive discussions on the design principles, structure–property relationships, and device architectures of porphyrin‐based non‐fullerene acceptors in molecular photovoltaics, the reader is referred to a previous review.^[^ ^86^ ^]^

**Figure 8:**
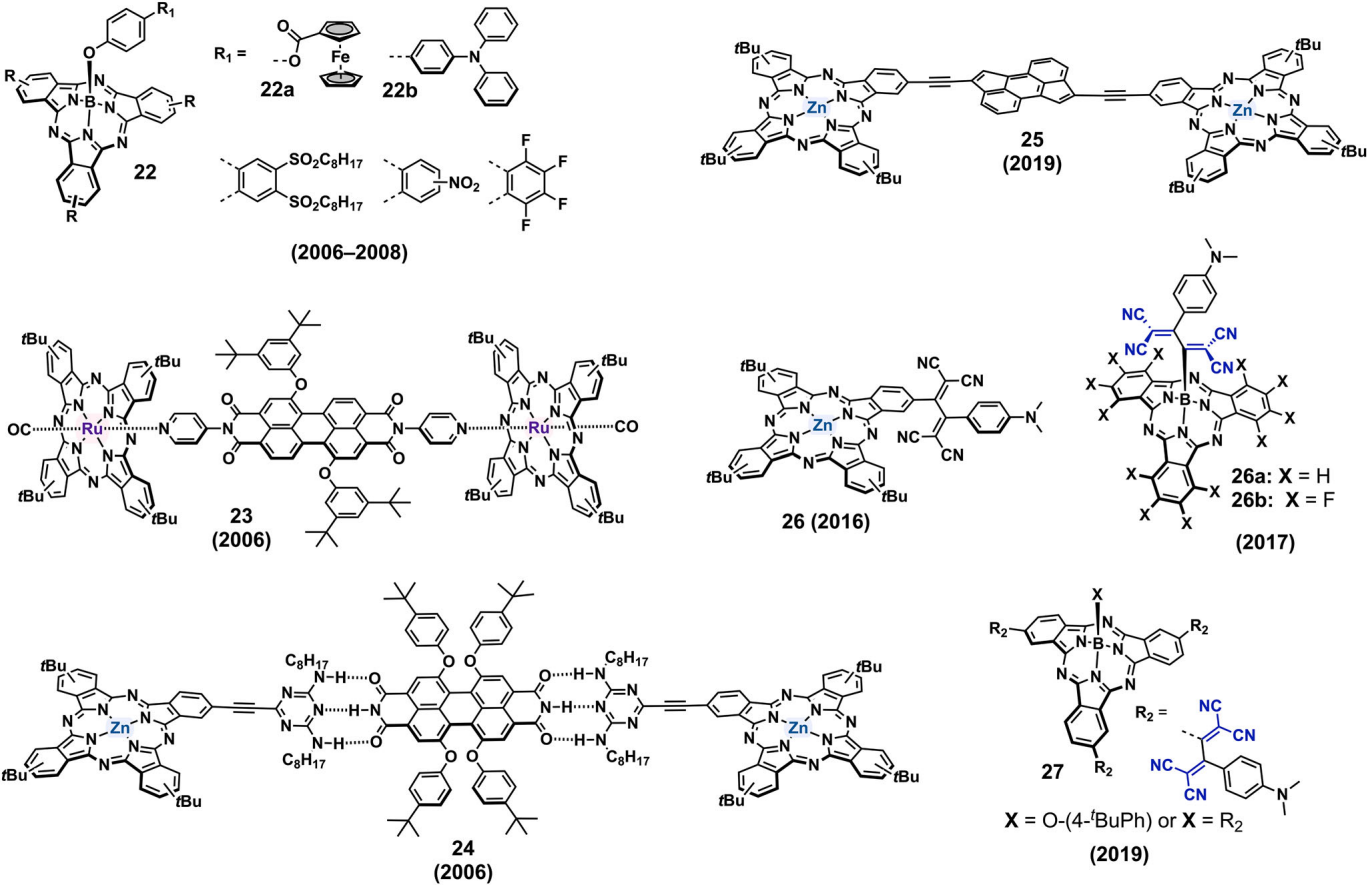
Representative azaporphyrinoid‐based supramolecular and covalent electron donor–acceptor systems employing non‐fullerene acceptors.

The collaborative investigations between Guldi and Torres groups aimed to develop new architectures with broader absorption profiles, enhanced redox tunability, and more defined photophysical behavior. SubPcs were identified as promising electron acceptors, especially when bearing electron‐withdrawing substituents. Notably, the first reported case of photoinduced intramolecular oxidation of C_60_ was achieved using a SubPc–C_60_ conjugate.^[^ ^73^ ^]^ Extending this concept, a series of SubPc–ferrocene (Fc) conjugates (**22a**; **Figure** **8**)^[^ ^87^ ^]^ with different electron‐withdrawing peripheral groups—fluoro, nitro, and alkylsulfonyl substituents—were prepared, revealing how substitution modulates their redox and photophysical behavior. All dyads exhibit reversible Fc‐based oxidation waves shifted positively by +0.2 to +0.3 V versus Fc/Fc⁺. The first one‐electron reduction potentials of the SubPc core are −1071 mV (fluoro), −1009 mV (nitro), and −883 mV (alkylsulfonyl) in THF, reflecting a stabilization of the LUMO with increasing electron‐withdrawing character. These electrochemical differences translate into marked variations in photophysical response. Fluorescence quantum yields decrease dramatically upon dyad formation, from ≈0.4–0.5 in the unsubstituted references to <0.01 in all dyads, with singlet excited‐state lifetimes reduced from ≈3 ns to ≈100 ps. Ultrafast transient absorption spectroscopy demonstrates rapid photoinduced electron transfer from the Fc donor to the SubPc acceptor. The fluoro‐substituted dyad shows a CS state lifetime of 29 µs, while the nitro‐substituted and alkylsulfonyl‐substituted dyads reach 55 and 231 µs, respectively. These long‐lived radical ion pair states—among the longest reported for dyads of this simplicity—are stabilized by axial spatial decoupling, poor orbital overlap, and structural reorganization involving the polar B─X bond of the SubPc core upon reduction.

After establishing SubPcs as efficient electron acceptors, a dodecafluoro‐substituted SubPc bearing a triphenylamine (TPA) unit at the axial position was synthesized and investigated (**22b**). Upon photoexcitation, ultrafast charge separation occurred from the SubPc to the TPA, as confirmed by femtosecond transient absorption spectroscopy.^[^ ^88^ ^]^ In this case, the radical‐ion pair lifetime ranges from 17 ps (toluene) to 9 ps (THF) and 7 ps (benzonitrile). In addition, a SubPc‐polymethine cyanine panchromatic light harvester was synthesized and characterized.^[^ ^89^ ^]^ In this D‐A conjugate, the complementary properties of heptamethine cyanine as a NIR light absorber and electron donor were combined with those of F_12_SubPc as a visible light absorber and electron acceptor, resulting in panchromatic absorption. Although no significant ground‐state interactions were observed between the two electroactive units, intramolecular electron transfer occurred in the excited state. The different singlet excited‐state energies of 2.2 eV for SubPc and 1.35 eV for cyanine, relative to the CS state energy of 1.1 eV, led to distinct charge separation dynamics of 5.7 and 550 ps, respectively, while the charge recombination dynamics remained unaffected at 215 ps.

Further contributions included the first detailed physicochemical characterization of SubPzs, homologues of SubPcs with higher axial reactivity.^[^ ^90^ ^]^ SubPzs exhibited stable oxidized and reduced forms, as well as luminescence with quantum yields comparable to SubPcs. Their photophysics proved highly sensitive to peripheral substitution, and intersystem crossing efficiently populated long‐lived triplet states, reinforcing their relevance for photovoltaic applications. This reactivity enabled the synthesis of axially bridged SubPz dimers and trimers using hydroquinone and phloroglucinol spacers.^[^ ^91^ ^]^ Organometallic functionalization of these arrays yielded ruthenoarenes,^[^ ^92^ ^]^ which provided additional control over ground‐ and excited‐state electronic communication.

In parallel, the strong electron‐accepting characteristics of PDIs made them attractive non‐fullerenic partners in D‐A assemblies with porphyrinoids. The first Pc‐PDI conjugates utilized axial coordination of pyridine‐substituted PDIs to ruthenium(II) Pcs, forming orthogonal triads with minimal electronic coupling and slow charge recombination kinetics (**23**; Figure 8).^[^ ^93^ ^]^ Extensions of this strategy yielded higher‐order assemblies, including PDI‐Ru(II)Pc pentads and Fc‐functionalized Pc‐PDI hybrids. These systems demonstrated tunable photophysical behavior, with electron or energy transfer pathways dictated by the metal center and substitution pattern.

Covalently linked Zn(II)Pc–PDI arrays with ethynyl bridges were also developed, affording electronically coupled systems that nonetheless exhibited unusually long‐lived CS states.^[^ ^94^, ^95^, ^96^, ^97^, ^98^ ^]^ Complementary approaches based on hydrogen bonding allowed for the formation of supramolecular Zn(II)Pc–PDI triads and Pc–PDI–Pc assemblies via melamine–imide recognition motifs (**24**; Figure 8).^[^ ^99^, ^100^ ^]^ The spatial arrangement dictated the occurrence of energy or electron transfer, with transient absorption confirming the presence of radical ion pair states in geometrically optimized configurations.

Additional conjugates featured SubPcs as donors and PDIs as acceptors, joined via B─N covalent bonds to promote ultrafast charge transfer.^[^ ^101^ ^]^ Reversed D‐A roles were also examined by introducing phenothiazine (PTZ) donors at the SubPc axial position. The versatility of these frameworks enabled their adaptation to diverse architectures, including Pcs conjugated to a wide array of electroactive units such as Ru(II)(bpy)_3_, anthraquinone, flavin, corannulene, oligo(*p*‐phenylenevinylene), and squaraine derivatives.^[^ ^102^, ^103^, ^104^, ^105^, ^106^, ^107^ ^]^ On the other hand, noncovalent assemblies between electron‐rich corroles and electron‐deficient Pcs offer a versatile platform for tuning photoinduced charge transfer processes.^[^ ^108^ ^]^ In particular, the axial coordination of a 10‐meso‐pyridine corrole to a Zn‐phthalocyanine affords stable donor–acceptor complexes with high binding constants and well‐defined supramolecular architecture. Spectroscopic studies reveal efficient formation of long‐lived CS states upon excitation, underscoring the potential of these systems in light‐harvesting and energy conversion applications, and highlighting key differences from covalently linked analogues. SubPc–Corrole dyads have also been used for the same purpose.^[^ ^109^ ^]^

Among the most compelling alternatives to fullerenes are cyclopenta‐fused polycyclic aromatic hydrocarbons (CP‐PAHs). Therefore, cyclopenta[hi]aceanthrylene (CPA) was employed as an electron acceptor in porphyrinoid‐based D‐A systems.^[^ ^110^ ^]^ CPA derivatives featuring electron‐donating ethynyl‐Zn(II)Pc (**25**; Figure 8) strong electronic coupling and unique charge‐transfer characteristics, including fluoro‐solvatochromism and quadrupolar‐to‐dipolar excited‐state evolution.^[^ ^111^ ^]^ These properties were confirmed by transient absorption spectroscopy, revealing solvent‐dependent stabilization of excited states and radical ion pairs.

Furthermore, CPA derivatives enabled the non‐covalent exfoliation of single‐walled carbon nanotubes (SWCNTs), as demonstrated by microscopy, Raman spectroscopy, and ultrafast spectroscopy.^[^ ^112^ ^]^ This exemplifies the multifunctionality of CPA‐based materials across different electronic environments.

TCBD derivatives hold a privileged position among the most versatile non‐fullerene acceptors.^[^ ^113^ ^]^ Therefore, the earliest design strategy explored by the two research groups in the TCBD field involved the covalent integration of one or four TCBD units into Zn(II) phthalocyanine (Pc) frameworks (**26**; Figure 8), in collaboration with the Diederich group.^[^ ^114^ ^]^ Comprehensive photophysical, electrochemical, and spectroelectrochemical analyses of two representative Pc–TCBD–aniline D‐A conjugates revealed broad panchromatic absorption profiles spanning the visible to near‐infrared (NIR) regions. These studies uncovered distinctive ground‐ and excited‐state behaviors. Notably, intense ground‐state charge transfer (CT) interactions between the electron‐rich Pc core and the TCBD acceptors were observed, establishing a previously unreported phenomenon in both Pc and TCBD‐based porphyrinoid chemistry.

Beyond Pc derivatives, covalent incorporation of TCBD units was extended to SubPcs.^[^ ^115^ ^]^ Specifically, SubPcs functionalized with TCBD‐aniline moieties at the axial position and bearing either hydrogen (**26a**) or fluorine atoms at the periphery (**26b**) were synthesized and structurally characterized by single‐crystal X‐ray diffraction. Each conjugate consisted of a racemic mixture of atropisomers, separated via chiral HPLC. Additional studies demonstrated that both atropisomers exhibit remarkable configurational stability, and a unique photoinduced racemization pathway enabled by triplet‐state activation was reported.^[^ ^116^ ^]^ Remarkably, the nature of the peripheral substituents in **26a** and **26b** was found to exert a decisive influence on the type of excited‐state interaction and deactivation pathway. In **26a**, spectroscopic evidence revealed ground‐state CT interactions between the SubPc macrocycle and the axial TCBD unit—an unprecedented observation in SubPc chemistry. Such interactions are proposed to facilitate efficient charge separation and suppress recombination by stabilizing CT intermediates, a feature particularly relevant for organic photovoltaic applications. In contrast, **26b** displayed markedly different behavior: the strong electron‐withdrawing fluorine substituents promoted the formation of an intramolecular exciplex between F_12_SubPc and the electron‐rich aniline, resulting in a broad and intense near‐infrared (NIR) emission. This exciplex emission, stemming from a polarized excited‐state CT interaction, was confirmed by time‐resolved absorption and fluorescence measurements. Notably, this finding represents the first report of exciplex formation in SubPc–TCBD systems, highlighting how subtle electronic tuning at the periphery enables access to distinct excited‐state manifolds and emission pathways.

To further investigate structure–property relationships, new SubPc derivatives were prepared bearing three or four TCBD‐aniline units at peripheral or combined axial/peripheral positions (**27**).^[^ ^117^ ^]^ Photophysical and electrochemical experiments revealed a substantial degree of electronic communication between the SubPc core, TCBD units, and aniline donors. Upon photoexcitation, charge transfer states involving SubPc donors and TCBD acceptors were identified. Interestingly, differences in excited‐state dynamics emerged depending on TCBD positioning: in the tri‐substituted SubPc, relaxation proceeded directly to the ground state, while the tetra‐substituted system formed a stabilized radical ion pair intermediate prior to deactivation.

In a more recent extension of this work, the Torres and Guldi groups, in collaboration with the Osuka group, synthesized and characterized two SubPs functionalized with a single TCBD‐aniline at either the meso or axial positions.^[^ ^118^ ^]^ Structural elucidation via X‐ray crystallography confirmed distinct conformations. The anchoring position of the TCBD unit significantly impacted the structural and electronic features of each conjugate. The compound with axial substitution, showed separable Ra and Sa enantiomers by chiral HPLC and displayed high configurational stability. In contrast, the one bearing peripheral substitution, could not be resolved into enantiomers due to enhanced conformational flexibility. From a physicochemical perspective, axial versus peripheral TCBD attachment modulated the HOMO–LUMO gap (1.77 eV for 40 vs 2.12 eV for 41), affecting photophysical behavior.

Later, two F_12_SubPcs with extended tetrathiafulvalene (TTF) moieties at their axial positions were prepared to investigate the impact of the distance between the electron donor and acceptor on photoinduced electron transfer.^[^ ^119^ ^]^ Steady‐state measurements revealed that, in the ground state, SubPc and extended TTF acted as independent entities, while transient absorption spectroscopy showed excited state interactions. Rapid charge separation resulted in a radical ion pair state with a lifetime influenced by the nature of the linker. Notably, adding an extra carbon between the electron donor and acceptor shifted the unwanted charge recombination dynamics from the normal to the inverted region of the Marcus parabola.

A different class of π‐extended near‐infrared materials was developed by fusing anthracene units to oligo‐BODIPYs via Au(I)‐catalyzed cyclization.^[^ ^120^ ^]^ The resulting fully conjugated systems exhibited tunable NIR absorption and emission, pronounced electron‐accepting character, and face‐to‐face supramolecular organization. Structural and optoelectronic properties were elucidated through spectroscopy, electrochemistry, and DFT calculations. These materials offer also a versatile platform for NIR‐active molecular optoelectronics.

Altogether, this research trajectory demonstrated a deliberate and strategic departure from traditional fullerene‐centered D‐A systems toward the development of structurally diverse and electronically tunable porphyrinoid‐based architectures featuring non‐fullerene acceptors (NFAs). Through molecular engineering, key design elements such as conjugation topology, substituent positioning, and donor–acceptor connectivity were optimized to modulate frontier orbital energies, redox potentials, and excited‐state dynamics. These rational modifications enabled precise control over ground‐ and excited‐state charge‐transfer processes, enhancing light absorption across the visible–NIR spectrum and facilitating long‐lived CS states. Functionally, the integration of NFAs broadened the scope of azaporphyrinoid materials beyond conventional photonic roles, enabling their implementation in next‐generation optoelectronic applications. As a result, this body of work established a versatile platform for tailoring photophysical and electrochemical properties through covalent linkage of azaporphyrinoids to π‐conjugated NFAs, offering new paradigms in the design of high‐efficiency light‐harvesting systems and organic electronic devices.

In summary, the integration of NFAs with azaporphyrinoids broadened spectral and redox properties, enabling the design of versatile photoactive systems beyond fullerene‐based frameworks.

## 3 Porphyrinoid–Carbon Hybrid Architectures: Conjugation with 1D and 2D Carbon Allotropes

The integration of porphyrinoids with 1D and 2D carbon allotropes has established a powerful strategy for constructing hybrid materials with enhanced optoelectronic function. CNTs and graphene provide exceptional conductivity, large surface area, and tunable electronic structures,^[^ ^121^ ^]^ while azaporphyrinoids such as Pcs offer strong absorption and redox activity. Covalent and noncovalent approaches were systematically applied to link these components, enabling controlled charge separation, directional energy flow, and electronic coupling. Synthetic modifications—ranging from click chemistry to dendritic substitution—allowed fine‐tuning of the donor–acceptor interface. These architectures support the development of advanced materials for photovoltaics, sensing, and molecular electronics.^[^ ^122^ ^]^

To harness the full potential of 1D and 2D nanocarbons, synthetic organic chemistry has served as a powerful tool to develop hybrid systems where electro‐ and photoactive Pcs are covalently or noncovalently linked to CNTs and graphene derivatives. Covalent attachment ensures kinetic stability and robust charge‐transfer pathways, whereas noncovalent interactions preserve the intrinsic electronic properties of the carbon scaffolds and enable reversible supramolecular architectures. Following successful advances in fullerene‐based D‐A conjugates, the transition toward CNT–Pc and graphene–Pc hybrids constituted a logical and strategic extension aimed at exploring new charge‐transfer regimes. In early pioneering work, the Torres group reported the synthesis of donor–acceptor conjugates in which Zn(II)Pcs were covalently linked to SWCNTs.^[^ ^123^ ^]^ At that time, SWCNTs had already been recognized for their electron‐accepting character, and efforts focused on grafting electron‐rich units—ranging from pyrene to ferrocene and porphyrins—onto their surfaces. However, no Pc‐based systems had yet demonstrated covalent sidewall functionalization of pristine SWCNTs or photophysical evidence of photoinduced charge transfer.

A breakthrough came with the use of 1,3‐dipolar cycloaddition of azomethine ylides, which had already proven effective for modifying the sidewalls of CNTs. This methodology was adapted to install Pcs on SWCNTs through a two‐step procedure: initial cycloaddition involving N‐octylglycine and 4‐formylbenzoic acid to generate carboxyl‐functionalized SWCNTs, followed by esterification with hydroxymethyl‐substituted Zn(II)Pc or H_2_Pc chromophores.^[^ ^124^ ^]^ These D‐A conjugates exhibited clear spectroscopic signatures of electron transfer, as confirmed by nanosecond transient absorption spectroscopy. Signals corresponding to the one‐electron oxidized Pc, along with bleaching of van Hove singularities in the SWCNTs, provided direct evidence for photoinduced charge separation.

**Figure 9:**
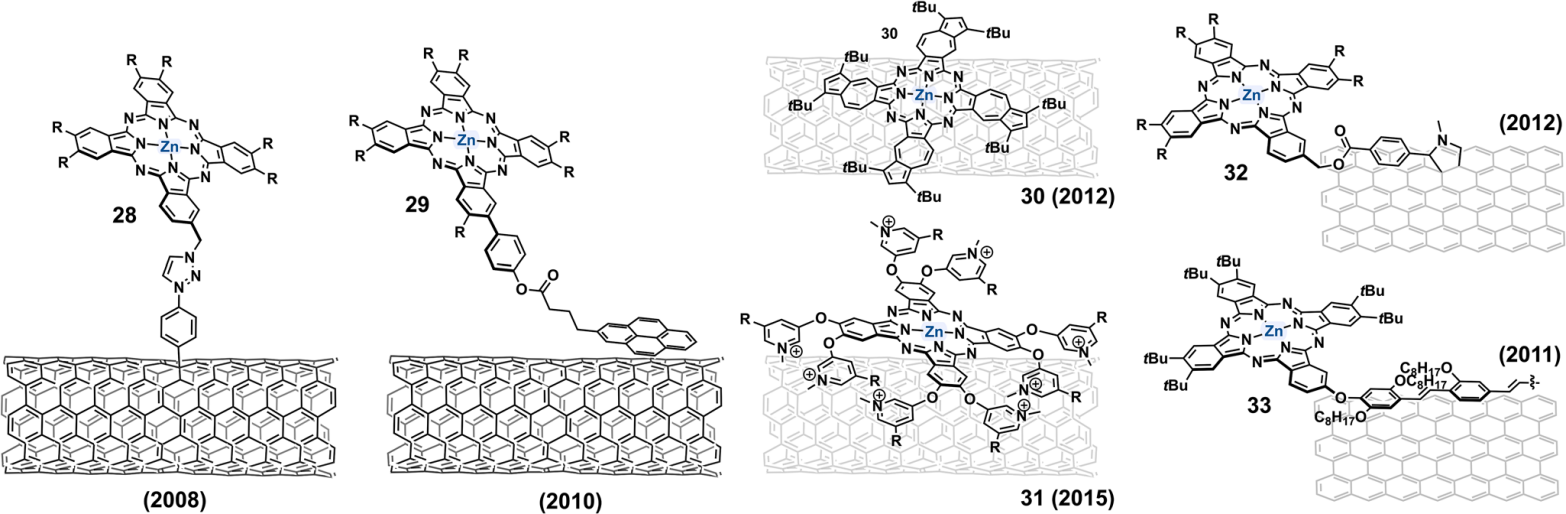
Structures of representative hybrid architectures formed via covalent or supramolecular linking of Pcs with 1D or 2D carbon allotropes.

A subsequent enhancement in synthetic efficiency and functionalization degree was achieved through the implementation of a copper‐catalyzed azide–alkyne cycloaddition (CuAAC) strategy.^[^ ^125^ ^]^ This click‐chemistry approach enabled the formation of the corresponding conjugate **28** (**Figure** **9**) via the reaction between an azide‐terminated Zn(II)Pc and ethynyl‐functionalized SWCNTs, yielding higher degrees of substitution and improved structural uniformity. Beyond the remarkable structural uniformity of **28**, its photophysical properties reveal highly efficient photoinduced charge separation. Upon selective ZnPc excitation (*λ*_exc_ = 660 nm), the characteristic singlet excited‐state features of ZnPc—transient maximum at 490 nm and ground‐state bleaching at 610–685 nm—decay rapidly within ≈20 ps, contrasting the ≈3.0 ns lifetime of the unbound ZnPc reference. Simultaneously, new absorption bands at 520 and 840 nm emerge, consistent with the formation of the ZnPc˙⁺ species, while blueshifts of the van Hove singularity bleaches in the NIR region (e.g., 963 → 940 nm) indicate electron injection into the SWNT conduction band.

A collaborative effort between the two groups led to significant advances in the noncovalent functionalization of SWCNTs with Pcs. Supramolecular immobilization was directed by π–π stacking, hydrophobic interactions, and van der Waals forces, providing structurally controlled D‐A interfaces while preserving the native electronic properties of the SWCNTs. This concept was applied to overcome the limited interaction efficiency in solution that Pc–SWCNT assemblies initially exhibited. Namely, oligo(ethylene glycol)‐substituted dendritic wedges were introduced onto hydrophobic Pc units, which enabled robust adhesion in both polar and aqueous environments and resulted in stable dispersions.^[^ ^126^ ^]^ Spectroscopic investigations revealed pronounced ground‐ and excited‐state coupling between the donor and acceptor components. Nanosecond transient absorption spectroscopy confirmed photoinduced charge separation, as evidenced by the formation of radical ion pairs.

Going one step further, inspired by prior studies had shown that aromatic systems such as pyrene and Pors exhibit a strong affinity for SWCNT surfaces and facilitate their dispersion in organic and even aqueous media when suitably functionalized with hydrophilic moieties, our groups reported an alternative modular strategy relied on conjugating anchoring groups such as pyrene or Pors to the periphery of Zn(II)Pcs (e.g., **29** in Figure 9).^[^ ^127^, ^128^ ^]^

In contrast to **28**, the noncovalent conjugation of ZnPc–pyrene conjugates to SWNTs via π–π interactions through the pyrene moiety was shown to provide efficient immobilization of the Pcs while preserving the electronic structure of the carbon scaffold. This association was spectroscopically evidenced by red‐shifted Q‐bands and nearly complete fluorescence quenching, consistent with strong excited‐state interactions. Ultrafast transient absorption studies revealed electron transfer from the phthalocyanine singlet excited state to the SWNTs on the picosecond timescale, leading to long‐lived CS states with decays extending beyond the 3.0 ns time window of the experimental setup. These photophysical dynamics translated into high device performance, as discussed in Section 4.1.

o‐PPV oligomers, which can wrap around nanotubes, were also explored as conjugated anchors.^[^ ^129^, ^130^ ^]^ When functionalized with pendant Zn(II)Pc units, these oligomers promoted non‐covalent interaction and enabled the formation of D‐A ensembles. Photophysical studies demonstrated that the interaction strength was governed by multiple factors, including the length of the conjugated backbone, the electron‐donating or ‐withdrawing character of the oligomer, and the spatial distance between Pc and the conjugated scaffold.

Further insight was provided by the use of azulenocyanines—Pc analogues bearing four di‐*tert*‐butylazulene units fused to a tetraazaporphyrin core (**30**, Figure 9).^[^ ^131^ ^]^ This derivative exhibits remarkable photophysical characteristics suitable for light‐harvesting applications. Its absorption spectrum is panchromatic, extending from 270 to 1150 nm, with intense maxima at 303 (ε = 96 000 L mol^−1^ cm^−1^), 403, 575, and a prominent NIR band at 1020 nm (ε = 58 000 L mol^−1^ cm^−1^), along with a shoulder at 940 nm. Fluorescence emission is centered at 1050 nm with a weaker band at 1250 nm upon 910 nm excitation, though the fluorescence quantum yield is very low (Φ ≈ 10^−^⁴). Electrochemical studies reveal three oxidation processes at +0.20, +0.50, and +1.01 V versus Fc/Fc⁺, with the first two being reversible. Upon oxidation, new absorption bands emerge at 345, 461, 520, 647, and 1140 nm, corresponding to the radical cation form. Importantly, its extended π‐surface allowed for highly effective adsorption onto SWCNTs, leading to stable nanohybrids. As a result, **30** exhibits significant red‐shifted and broadened absorption bands (e.g., the 1020 nm band shifts by 45 nm), and complete fluorescence quenching, indicative of strong ground‐state interactions and efficient charge transfer. Femtosecond transient absorption spectroscopy confirms ultrafast photoinduced electron transfer to SWNTs, with charge separation lifetimes of ≈124 ps.

While Pcs typically serve as electron donors and SWCNTs as electron acceptors, role inversion was also achieved. Through peripheral functionalization with electron‐withdrawing pyridyloxy groups, Zn(II)Pcs were converted into electron‐accepting units (**31**, Figure 9).^[^ ^132^ ^]^ The noncovalent hybridization of this electron‐accepting Pc yielded water‐processable supramolecular systems that exhibited efficient photoinduced charge separation. Upon photoexcitation at 387 nm, transient absorption spectroscopy revealed the formation of ZnPc radical anions, evidenced by maxima at 585 nm and bleaching at 690 nm, corresponding to reduced ZnPc and oxidized SWNT species, respectively. The hybrid system displayed markedly accelerated excited‐state dynamics, with biexponential decay components of 0.5 and 14 ps, compared to 0.9 and 215 ps for SWNTs stabilized by sodium dodecylbenzenesulfonate (SDBS). Steady‐state absorption spectra showed a redshift of the ZnPc Q‐band from 677 to 692 nm upon hybrid formation, indicative of strong π–π interactions. Fluorescence emission of the SWNTs was strongly quenched in the hybrid, and the fluorescence quantum yield of this electron‐accepting ZnPc was reduced to 0.22, relative to 0.30 for the reference ZnPc, consistent with efficient electron transfer from the electron‐donating SWNTs to the cationic ZnPc.

Following its isolation, graphene rapidly gained attention as a versatile 2D carbon platform with superior electronic, mechanical, and thermal performance.^[^ ^133^ ^]^ Its high surface area and low‐cost production via liquid‐phase exfoliation of graphite rendered it attractive for optoelectronic applications, including its combination with photoactive molecules such as Pcs.

Despite its excellent properties, pristine graphene is considerably less reactive than SWCNTs or fullerenes due to the absence of significant strain in its basal plane. Nevertheless, functionalization strategies developed for other carbon allotropes, including 1,3‐dipolar cycloaddition chemistry, were successfully adapted for use on graphene with varying yields. At the time, only Pc conjugates with graphene oxide (GO) had been reported.^[^ ^134^ ^]^ The Guldi and Torres groups reported the first covalent functionalization of the basal plane of few‐layer graphene (FLG), prepared by liquid‐phase exfoliation, using a two‐step protocol. This involved 1,3‐dipolar cycloaddition between N‐methylglycine and 4‐formylbenzoic acid, followed by esterification with an alcohol‐terminated Pc (**32**; Figure 9).^[^ ^135^ ^]^ The resulting hybrid exhibited efficient photoinduced electron transfer and prolonged charge separation lifetimes, supporting its suitability for photovoltaic and optoelectronic applications. Steady‐state absorption spectra of **32** in DMF revealed broadened and bathochromically shifted Q‐band maxima at 673 and 733 nm, indicating strong electronic coupling between the Pc and the graphene basal plane. Upon excitation at 387 nm, femtosecond transient absorption spectroscopy of **32** showed rapid decay of Pc singlet excited states within 5 ps, alongside the emergence of new transient features at 515 and 850 nm, characteristic of the one‐electron oxidized Pc species. Simultaneously, a broad signal at 1100 nm developed in the NIR, attributed to conduction band electrons injected into graphene. Kinetic analysis revealed biexponential dynamics with charge separation and recombination lifetimes of (3.3 ± 0.5) and (270 ± 10) ps in DMF, and slightly longer recombination in NMP at (340 ± 10) ps. Fluorescence of the Pc was entirely quenched in hybrid **32**, confirming the ultrafast photoinduced charge separation process. This work represented the first report of a covalent Pc–graphene hybrid based on liquid‐phase exfoliated graphene and demonstrated that covalent grafting preserved graphene's electronic properties while enabling robust photochemical functionality.

A major focus of the collaborative efforts was also placed on the non‐covalent exfoliation and functionalization of graphene using phthalocyanine (Pc)‐based architectures. The initial strategy involved the application of Zn(II)Pc–*o*‐PPV conjugates (**33**; Figure 9), where the conjugated oligomer backbone supported both the stabilization of exfoliated graphene layers and the dispersion of graphite.^[^ ^136^, ^137^ ^]^ In contrast, Zn(II)Pc alone proved ineffective for promoting significant graphite exfoliation under identical conditions. Systematic variation of the *o*‐PPV backbone—both in length and in p/n‐type character—revealed that extended chains with strong π‐conjugation led to enhanced graphene interaction and more efficient exfoliation. Upon hybrid formation, the ZnPc Q‐band absorption exhibited a 32 nm bathochromic shift from 675 to 707 nm, indicative of π–π stacking and electronic communication between ZnPc and graphene. Fluorescence studies revealed nearly complete quenching of ZnPc emission in hybrid **33**, with a quenching efficiency exceeding 60%, while the fluorescence lifetime of the free ZnPc oligomer (3.1 ns) remained unchanged but was drastically reduced in intensity due to the predominance of nonfluorescent hybridized species. Femtosecond transient absorption spectroscopy following excitation at 387 and 700 nm demonstrated efficient photoinduced electron transfer from ZnPc to graphene. This was evidenced by the formation of oxidized ZnPc at 840 nm and reduced graphene at 1290 nm, with ultrafast charge separation (*k*_CS_ = 7.1 × 10^11^ s^−1^) and recombination lifetimes of 360 ps (*k*_CR_ = 2.7 × 10⁹ s^−1^).

Additional molecular design strategies were implemented to enhance graphene binding. Substitution of the Pc macrocycle with pyrene moieties resulted in strong π–π interactions with graphene, facilitating the formation of stable dispersions.^[^ ^138^ ^]^ Likewise, Newkome‐type dendritic wedges functionalized with terminal carboxylate groups, when introduced at the Zn(II)Pc periphery, promoted aqueous compatibility and efficient supramolecular association with graphene.^[^ ^139^ ^]^ Structural modification of the Pc itself, using azulenocyanine derivatives with extended aromatic surfaces, also enabled the production of high‐quality few‐layer graphene dispersions and stable donor–acceptor ensembles.^[^ ^140^ ^]^

The degree of electronic communication between Zn(II)Pc and graphene in both ground and excited states was rigorously examined. Typically, Pcs act as electron donors upon photoexcitation. However, targeted peripheral functionalization with electron‐withdrawing groups reversed this behavior, rendering the Pc units electron‐accepting. In both covalently and noncovalently assembled systems, charge transfer from graphene to the excited Pc was spectroscopically confirmed.^[^ ^141^, ^142^ ^]^ Transient absorption measurements revealed the formation of one‐electron reduced Zn(II)Pc species alongside a broad bleaching in the graphene absorption spectrum, indicative of hole generation in the valence band and the formation of well‐defined CS states.

In summary, azaporphyrinoid–carbon nanohybrids were constructed through modular covalent and supramolecular strategies, enabling tunable charge transfer and optoelectronic properties tailored to energy conversion applications.

## 4 Azaporphyrinoid‐Based Systems in Energy Conversion Applications

Azaporphyrinoids have garnered significant attention as multifunctional chromophores in emerging solar energy technologies. Their robust chemical architecture, synthetic tunability, and favorable optoelectronic characteristics—particularly in charge separation and light harvesting—render them ideal candidates for integration into photovoltaic and photonic applications. This section examines the evolution of azaporphyrinoid‐based systems in the context of molecular photovoltaics and singlet fission, highlighting the design principles underpinning their functional implementation. It is important to note that, while fullerenes are initially discussed as prototypical electron acceptors in molecular D‐A systems (Sections 2.1 and 2.2), the aim of Section 4.1 is to examine the role of azaporphyrinoids as electroactive components in photovoltaics. Accordingly, the focus is placed on the azaporphyrinoid unit and its integration with carbon nanostructures and metal oxides in device‐relevant settings

### 4.1 Azaporphyrinoids in Molecular Photovoltaics

Functionalized azaporphyrinoids have emerged as key components in advanced energy conversion systems, owing to their structural versatility, photochemical stability, and ability to mediate electron transfer processes. This section highlights progress in the design and application of azaporphyrinoids within molecular photovoltaic architectures, with a focus on innovative strategies that enhance solar light harvesting and utilization. Representative advances stemming from the collaboration between the Torres and Guldi groups are showcased, underscoring their significant contributions to the field through the development of supramolecular assemblies and functional materials with optimized photoelectronic properties.

Following extensive investigations into photon and charge management in D‐A systems, azaporphyrinoid‐based materials were next evaluated in solar energy conversion schemes.^[^ ^143^ ^]^ Initial efforts focused on hybrid materials comprising Pcs and advanced 1D or 2D carbon allotropes. Several of these nanoconjugates were integrated into photoelectrochemical cells to explore their viability as photoactive components in functional devices.

Dye‐sensitized solar cells (DSSCs) served as the primary testbed,^[^ ^144^ ^]^ with photoanodes typically constructed from TiO_2_ films modified with a photosensitizer (PS), paired with a counter electrode and redox mediator. Pc–SWCNT and Pc–graphene D‐A conjugates were incorporated into photoanodes to assess electron injection capabilities. The covalent Zn(II)Pc–SWCNT conjugate **28** demonstrated a clear photocurrent onset above 0.4 V, attributed to electron injection into indium tin oxide (ITO), and delivered an internal photoconversion efficiency (IPCE) of 17.3% at +0.6 V and 385 nm.^[^ ^125^ ^]^

Motivated by these results, further Zn(II)Pc–SWCNT hybrids were evaluated. Noncovalent D‐A ensembles were prioritized to preserve SWCNT electronic properties and enhance charge transport. Thus, photoelectrochemical cells incorporating **29** displayed internal photoconversion efficiencies of up to 15% under short‐circuit conditions, increasing to 23% under mild positive bias (+0.1 V).^[^ ^145^ ^]^ In contrast, assemblies using Zn(II) azulenocyanines achieved only ≈1.9% and ≈2.6% IPCE, despite broader absorption profiles.^[^ ^48^ ^]^ Collectively, these findings positioned phthalocyanine–pyrene/SWNT hybrids among the most efficient noncovalently assembled donor–acceptor systems based on carbon nanotubes.

Water‐dispersible Zn(II)Pc–SWCNT nanohybrids enabled device fabrication under green processing conditions. Photoelectrodes were prepared by spraying aqueous solutions onto fluorine‐doped tin oxide (FTO) substrates and combined with Cu_2_S counter‐electrodes. Though IPCE values were modest (≈0.6%), the study validated aqueous‐phase device assembly and proof‐of‐concept operation.^[^ ^146^ ^]^ Encouraged by these results with SWCNT, Pc–graphene composites were introduced. The photoelectrochemical potential of **33** (Figure 9) was thus validated through solar cell prototypes, which achieved a sheet resistance of 90 kΩ □^−1^ at 75% transmittance and an incident photon‐to‐current IPCE of up to 0.3%, demonstrating the hybrid's suitability for optoelectronic applications.^[^ ^137^ ^]^ Similarly, azulenocyanine–graphene hybrids were incorporated into DSSC configurations as blocking layers.^[^ ^147^ ^]^ All these experiments established the functional integration of Pc–carbon D‐A materials into photoanodes.

Subsequently, attention turned to inverted or p‐type DSSCs.^[^ ^148^ ^]^ Unlike n‐type cells, p‐DSSCs operate via hole injection from a p‐type semiconductor (e.g., NiO) to the PS. Despite the conceptual advantage of p/n‐tandem configurations, performance remains limited by the poor efficiencies of p‐DSSCs, largely due to rapid recombination events. Push–pull PSs with spatially separated donor and acceptor units offer a rational design strategy.^[^ ^149^ ^]^ In this context, CuO nanostructures emerged as an alternative to NiO due to improved conductivity and charge mobility. The integration of Zn(II)Pcs into CuO photocathodes required modification of the macrocycle: three isoindole sites were functionalized with electron acceptors, and one with a COOH anchor. Branched sulfonyl groups suppressed aggregation and enhanced acceptor strength. Two anchoring motifs—direct COOH and carboxyethynyl—were evaluated.^[^ ^150^ ^]^ Electrochemical analyses (including Kelvin probe microscopy) indicated linker‐dependent energy level alignment. With an I^−^/I_3_^−^ electrolyte, the carboxyethynyl derivative yielded superior performance (*V*_OC_ and *J*_SC_), with PCEs of 0.103%. IPCEs peaked at 27.4% for the same compound at 670 nm. Electrochemical impedance spectroscopy supported better injection and transport in the carboxyethynyl compound. Replacing the electrolyte with [Co(dtb‐bpy)_3_][PF_6_]_2_/_3_ improved PCEs to 0.191%. Although efficiencies remained low, they exceeded prior CuO‐based systems by nearly an order of magnitude. Optimization of mesoporous CuO electrodes included control of calcination conditions, film thickness, and electrolyte composition.^[^ ^151^ ^]^ Precipitation methods refined nanoparticle size and increased surface area. Electrolyte tuning using solvents, ionic liquids, and additives further enhanced PCE to 0.156%.

Subsequent efforts targeted PS engineering. Spacers—ethynylphenyl and ethynyl‐TPA—were inserted between the Zn(II)Pc core and COOH anchor to enhance push–pull character and interfacial charge separation.^[^ ^152^ ^]^ Photocathodes employed optimized CuO nanoparticles^[^ ^153^ ^]^ and the I^−^/I_3_^−^ electrolyte.^[^ ^154^ ^]^ Despite lower *J*_sc_ due to reduced extinction coefficients, these new compounds achieved higher fill factors, likely due to suppressed recombination via increased donor–semiconductor separation. Device with the compound with spacer ethynyl‐TPA, with its TPA donor proximal to CuO, was integrated into tandem DSSCs using N719/TiO_2_ photoanodes. The best device yielded 0.69% efficiency, marking the first demonstration of CuO‐based tandem DSSCs.

Recent research focused on the advancement of selective near‐infrared DSSCs, identified as a promising technology for building‐integrated photovoltaics. These systems combine energy generation with visual transparency and aesthetic adaptability. Through molecular engineering of sensitizers, selective absorption in the NIR region—representing up to 45% of the solar spectrum—is achieved without affecting visible light transmission. Recent efforts addressed the development of colorless, transparent DSSCs by integrating NIR‐absorbing dyes with optimized photoanodes, redox mediators, and counter electrodes. The interplay among these components proves essential to balance device efficiency and visual appeal. A recent review provides an overview of the material strategies and implementation challenges associated with bringing NIR‐selective DSSCs closer to architectural applications.^[^ ^155^ ^]^

Recent efforts have been directed toward the development of perovskite‐sensitized solar cells, with Pcs serving as hole‐transporting materials, reaching PCEs up to 20.0%.^[^ ^156^, ^157^ ^]^ Other works have been focused on elucidating the energy transfer mechanisms at metal halide perovskite–phthalocyanine interfaces. In this context, CsPbBr_3_ nanocrystals (NCs) were coupled with carboxylated ZnPc dyes to form nanohybrid assemblies. This study revealed that energy transfer proceeds via an atypical Dexter‐type singlet pathway, enabling efficient singlet oxygen generation. A combination of femtosecond‐to‐microsecond time‐resolved spectroscopies and global target analysis uncovered a complex excitonic landscape within the nanocrystals, which drives unidirectional energy transfer to ZnPc. Nearly quantitative singlet oxygen production is observed under selective perovskite excitation. These findings offer valuable insight into interfacial energy transfer dynamics and highlight the potential of perovskite–phthalocyanine systems in light‐driven photonic applications.

The Torres and Guldi groups, with Galan and Pérez‐Prieto group, uncovered the energy transfer mechanism at CsPbBr_3_ perovskite–phthalocyanine interfaces. They demonstrated that CsPbBr_3_ NCs coupled to carboxylated ZnPc enable near‐quantitative singlet oxygen photosensitization via an unusual Dexter‐type singlet energy transfer. Ultrafast‐to‐microsecond spectroscopy identified three excitonic NC components driving the process. These findings position CsPbBr_3_@ZnPc as a benchmark nanohybrid for photon harvesting and energy conversion, highlighting its promise as a versatile photosensitizer.^[^ ^158^ ^]^

π‐Extended azasubporphyrinoids were investigated as electron‐transporting materials (ETMs) in perovskite solar cells, in collaboration with Nazeeruddin.^[^ ^159^ ^]^ Subnaphthalocyanines (SubNcs) and subphthalocyanine dimers (SubPc_2_) were vacuum‐deposited as thin interlayers, facilitating efficient electron extraction at the perovskite/ETM interface. Device architectures incorporating these materials showed reduced hysteresis, improved charge transport, and enhanced operational stability. The extended π‐conjugation and tailored electronic structure of the subporphyrinoids played a decisive role in modulating interfacial energetics and charge dynamics. These results demonstrate the potential of π‐extended SubNcs and SubPc_2_ as effective ETMs for robust and stable perovskite‐based optoelectronic applications.

On the other hand, axially substituted SubPcs bearing chlorine or fluorine were integrated into devices fabricated by solution processing (CsFAMAPbIBr) and thermal evaporation (MAPI).^[^ ^160^ ^]^ Photoluminescence and time‐resolved spectroscopy revealed improved charge extraction and interfacial quality, particularly for chlorine‐substituted derivatives. The best‐performing architecture combined SubPc with C_60_ in a bilayer ETM, achieving a power conversion efficiency of 10.8%. These results demonstrate the suitability of SubPcs as functional electron transport materials in both solution‐processed and vacuum‐deposited PSCs.

The electron‐transporting properties of SubPcs were subsequently modulated to enhance the performance of inverted perovskite solar cells.^[^ ^161^ ^]^ Peripheral halogenation with chlorine and fluorine allowed fine‐tuning of interfacial energetics and film morphology. Photoluminescence and time‐resolved PL measurements revealed reduced charge recombination and more efficient electron extraction. Devices integrating optimized SubPc interlayers exhibited improved operational stability and reached a maximum power conversion efficiency of 13.6%. These findings highlight the critical role of molecular design in governing charge transport and establish SubPcs as promising non‐fullerene ETMs for stable p‐i‐n perovskite architectures.

In summary, azaporphyrinoids have been—and continue to be—implemented in dye‐sensitized and perovskite solar cells, facilitating electron extraction, spectral tuning, and enhanced device stability. In this regard, their integration with nanocarbons and metal oxides enabled tailored interfacial properties, whereas novel functionalization strategies proved essential to performance optimization.

### 4.2 Azaporphyrinoids for Singlet Fission and Related Photophysical Processes

The use of azaporphyrinoids as light‐harvesting antennas in SF systems has enabled complementary spectral absorption and directional energy transfer. Their tunable emission profiles and extended π‐systems facilitate intramolecular Förster resonance energy transfer (i‐FRET) to pentacene dimers, enhancing triplet generation. This subsection outlines molecular designs that modulate chromophore geometry, dipole alignment, and coupling strength. Covalent and supramolecular strategies are discussed, highlighting advances toward efficient SF sensitization in solution‐processable architectures.

**Figure 10:**
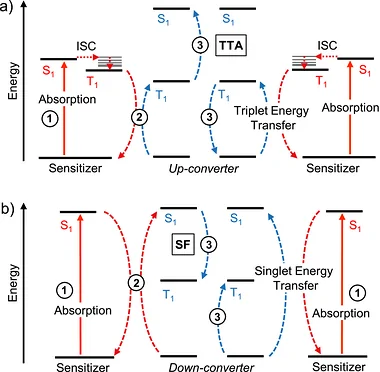
Schematic illustration of the operating principles of a) triplet–triplet annihilation (TTA‐UC) and b) singlet fission (SF). Red and blue arrows indicate processes occurring at the sensitizer and the converter, respectively. The temporal sequence of each step is indicated by numbered circles.

Maximizing solar spectrum utilization remains a central goal in the development of next‐generation photovoltaic technologies. On this basis, up‐ and down‐conversion strategies—commonly referred to as TTA‐UC and SF, respectively—are being extensively investigated to harness both high‐ and low‐energy photons that are typically not utilized by conventional semiconductors (**Figure** **10**).

TTA‐UC refers to a process in which two identical molecules in their triplet excited states (i.e., up‐converters) interact to produce one molecule in the singlet excited state and another in the ground state. In contrast, SF involves a singlet excited molecule interacting with a ground‐state partner (i.e., down‐converters) to generate two lower‐energy triplet excitons. In both processes, a sensitizer enhances the panchromatic response by transferring energy to populate either the singlet or triplet states of the converter. While sensitization of TTA‐UC using porphyrinoids has been widely developed over the years, our groups have filled the gap in down‐conversion by introducing the concept of sensitized singlet fission.

In this context, pentacene has been extensively studied as a benchmark SF material, exhibiting near‐unity triplet yields.^[^ ^162^ ^]^ However, its absorption is primarily confined to the 575–700 nm region, leaving the high‐energy (400–575 nm) portion of the spectrum underutilized.

To address this spectral gap, pentacene (Pnc) derivatives have been coupled to complementary light‐harvesting antennae capable of transferring energy via i‐FRET, thereby enhancing intramolecular SF (i‐SF) efficiency. The performance of such donor–acceptor systems depend critically on the spectral overlap between energy donor emission and energy acceptor absorption. However, the rational development of donor chromophores with tailored optical properties typically requires iterative synthesis and detailed photophysical characterization of both the individual components and the resulting conjugates.

**Figure 11:**
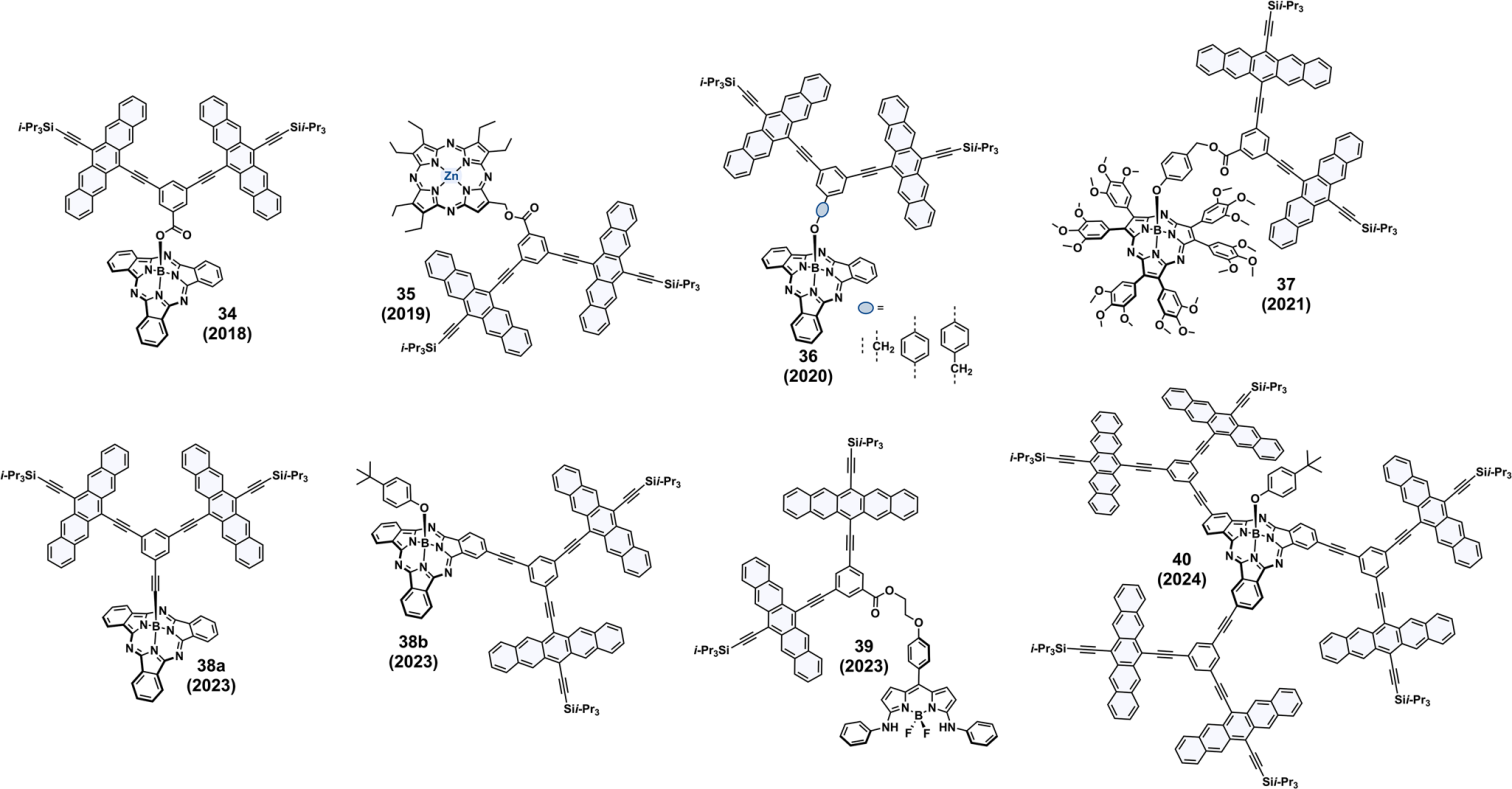
Molecular structures of porphyrinoid–pentacene dimer systems designed for sensitized singlet fission.

In this context, Guldi and Torres, in collaboration with Tykwinski,^[^ ^163^ ^]^ reported a series of covalently linked SubPc–Pnc_2_ conjugates (**34**; **Figure** **11**), designed to combine spectral complementarity with directional energy flow. The optimized overlap between SubPc emission and Pnc_2_ absorption enabled effective panchromatic light harvesting, extending up to ≈700 nm. This spectral coverage was functionally translated into directional i‐FRET from the SubPc antennae to the Pnc_2_ core, where efficient i‐SF yielded triplet excitons with quantum yields approaching unity.

Expanding the framework of synergistic light harvesting, peripherally alkylated Zn(II) porphyrazines (Zn(II)Pzs) were employed as complementary absorbers to Pnc_2_ (**35**).^[^ ^164^ ^]^ In these Zn(II)Pz–Pnc_2_ conjugates, the selection of the Pz scaffold was driven by two key design criteria. First, Zn(II)Pzs exhibit strong absorption in the 580–600 nm range, minimizing spectral overlap with the Pnc_2_ unit. Second, the peripheral attachment of Pnc_2_ to the Zn(II)Pz macrocycle allowed fine control over the donor–acceptor geometry, directly impacting i‐FRET efficiency. Compared to SubPc‐based analogues, this configuration introduced distinct electronic coupling pathways. The resulting assemblies displayed broadband (panchromatic) absorption and highly efficient intramolecular singlet fission, with triplet quantum yields approaching 200%.

A more recent study explored the synergistic integration of panchromatic absorption and i‐SF in a series of SubPc–Pnc_2_ conjugates (**36**), in which the molecular spacer was systematically varied in length and rigidity.^[^ ^165^ ^]^ By adjusting the number of aryl units and selectively inserting methylene groups, i‐FRET was modulated efficiently without modifying the SubPc donor unit. AM1–CIS calculations supported the experimental findings, confirming that spacer architecture plays a critical role in regulating both i‐FRET and i‐SF dynamics. The incorporation of a single phenyl ring led to an ≈3.8‐fold slowdown in i‐FRET and an ≈1.6‐fold reduction in i‐SF rates. This deceleration was attributed to quinonoid conjugation, which extended the acceptor orbital over both pentacene units and the bridging aryl group, thereby altering the electronic coupling landscape.

To further exploit panchromatic light harvesting and enable efficient triplet generation, SubPzs^[^ ^166^ ^]^ was also strategically conjugated to a pentacene dimer. Thanks to complementary absorption bands between 450 and 600 nm, the selected hexarylated SubPz was found to serve as an efficient energy acceptor and a SF platform (**37**).^[^ ^167^ ^]^ In this donor–acceptor architecture, i‐FRET facilitates highly efficient excitation energy delivery from the SubPz to the pentacene dimer. Upon energy transfer, the dimer undergoes i‐SF, generating a correlated triplet pair via a spin‐allowed intermediate. A hallmark of this system is the pronounced solvatochromic fluorescence of the SubPz, which shifts by up to 20 nm in polar solvents. This tunability enables dynamic modulation of the spectral overlap with the dimer's absorption, directly impacting both the Förster rate constant and the resulting triplet quantum yield. Xylene offers optimal conditions, yielding a Förster rate constant of 3.52 × 10^11^ s^−1^ and an exceptional triplet quantum yield of 171% ± 10%. Critically, the solvent‐dependent behavior intrinsic to SubPzs provides a powerful lever for fine‐tuning excited‐state interactions—without the need for structural modification—thus offering a streamlined route to maximize energy transfer efficiency and triplet generation.

More recently, two simple SubPc‐Pn_2_ SF systems were designed to evaluate the influence of chromophore orientation on energy transfer and fission efficiency.^[^ ^168^ ^]^ Two conjugates were synthesized (**38a–b**), each consisting of a pentacene dimer (serving as the SF unit) tethered via a rigid alkynyl bridge to a SubPc sensitizer, arranged either axially or peripherally. Steady‐state and time‐resolved spectroscopic analyses confirmed that both conjugates function as intended, displaying near‐unity energy transfer efficiencies and high triplet quantum yields via SF. Remarkably, energy transfer in the peripheral conjugate occurred ≈26 times faster than in the axial analogue, despite an ≈3 Å longer interchromophore distance. Theoretical calculations indicated that this rate enhancement arises from a more favorable orientation of the transition dipole moments. The peripheral arrangement showed a significantly larger dipolar coupling and orientation factor in the axial configuration. These findings underscore the critical role of chromophore geometry in governing energy transfer dynamics and optimizing SF sensitization strategies.

Building on previous donor–acceptor architectures, a new conjugate combining a 4,4‐difluoro‐4‐bora‐3a,4a‐diaza‐s‐indacene (BODIPY) donor with a pentacene dimer (Pnc_2_) was developed to enable broadband solar absorption and high triplet generation efficiency (**39**).^[^ ^169^ ^]^ The resulting BODIPY–Pnc_2_ construct absorbs across a wide spectral range and operates via a sequential energy transfer–singlet fission mechanism. Upon excitation, the singlet energy of the BODIPY unit is funneled to the pentacene core through i‐FRET, followed by efficient intramolecular singlet fission within the dimeric acceptor. This cascade generates a correlated triplet pair via a spin‐allowed intermediate. Solvent polarity emerges as a critical parameter for modulating both energy transfer and fission efficiency. The highest FRET rate constant (7.46 × 10^11^ s^−1^) and triplet quantum yield (207 ± 20%) are reached in benzonitrile, highlighting the strong sensitivity of the system to its dielectric environment. These results demonstrate the viability of BODIPY‐based donors in singlet fission platforms and offer a modular strategy for optimizing light‐harvesting performance without chemical modification of the core units.

Finally, to overcome the longstanding limitation of efficient free triplet generation in covalent singlet fission systems, a supramolecular strategy was devised to replicate solid‐state‐like triplet diffusion within a discrete molecular framework. While covalently linked chromophores in solution allow precise control over electronic coupling—essential for fast ^1^(T_1_T_1_) formation—this same confinement often leads to rapid triplet–triplet annihilation, hindering the release of free triplets. In contrast, entropy‐driven diffusion in the solid state enables spatial separation of triplets, improving overall yield, but molecular packing constraints make tuning of coupling inherently challenging. To bridge this gap, a hexameric architecture (HexPnc) was developed by covalently attaching three pentacene dimers to a central SubPc scaffold (**40**).^[^ ^170^ ^]^ This configuration promotes intramolecular cluster formation and facilitates entropic triplet migration within the molecular assembly, effectively emulating the diffusive separation typically afforded in the solid state. Transient absorption spectroscopy, combined with steady‐state optical studies and theoretical modeling, revealed enhanced triplet mobility and efficient dissociation of the correlated ^1^(T_1_T_1_) state. As a result, HexPnc achieved up to a 14‐fold increase in free triplet yield compared to the corresponding dimeric reference (DiPnc).

All in all, this design establishes a modular platform for reconciling strong interchromophore coupling with efficient triplet separation, offering critical insight for the development of next‐generation high‐performance singlet fission materials. As a summary of Section 4, azaporphyrinoid‐based systems were strategically designed for energy conversion applications by exploiting their structural diversity, redox flexibility, and photochemical robustness. Covalent and supramolecular approaches enabled precise control over charge and energy transfer processes. These architectures proved effective across a range of optoelectronic platforms, including molecular photovoltaics and singlet fission systems.

## 5 Conclusion

Taken together, these findings highlight the dual scientific and technological relevance of our work, situated at the intersection of fundamental discovery and functional design. Progress in solar energy conversion critically depends on both curiosity‐driven basic research and solution‐oriented applied development. Within the realm of advanced energy materials, understanding how light is absorbed, transformed, and transported at the molecular level provides the foundation for rational design. Our investigations into Pcs, SubPcs, and advanced carbon nanostructures exemplify this approach, addressing key questions surrounding photon harvesting, exciton generation, and charge separation with the goal of uncovering guiding design principles.

In these systems, fundamental studies elucidated the mechanisms that govern singlet fission, energy transfer, and triplet diffusion, offering valuable insight into the structure–function relationships that dictate photophysical behavior. Although this knowledge is not initially pursued with commercial objectives, it forms the basis for transformative advances in material performance. Indeed, once these principles are understood, they can be translated into practical innovations.

On the applied side, these same materials are evaluated under conditions relevant to device integration, with clearly defined metrics such as triplet yield, Förster transfer rate, or stability under operational conditions. Here, the focus shifts from why and how to when and how well—bridging fundamental understanding with tangible performance. SubPcs and carbon‐based materials show promise for deployment in optoelectronic devices, including photovoltaics and photodetectors.

In this context, the dual nature of our research—advancing fundamental knowledge while informing materials engineering—underscores the importance of a synergistic approach. The results presented here not only address open questions in photon and charge management but also offer a blueprint for the design of next‐generation functional materials in solar energy conversion.

In summary, by bridging mechanistic understanding with targeted material design, this work contributes a unified framework for the development of high‐efficiency energy conversion systems, where fundamental insight translates directly into technological opportunity.

## Conflict of Interest

The authors declare no conflict of interest.
